# Fluorinated maleimide-substituted porphyrins and chlorins: synthesis and characterization

**DOI:** 10.3762/bjoc.15.263

**Published:** 2019-11-13

**Authors:** Valentina A Ol’shevskaya, Elena G Kononova, Andrei V Zaitsev

**Affiliations:** 1 A. N. Nesmeyanov Institute of Organoelement Compounds of Russian Academy of Sciences, 119991, Vavilova St. 28, Moscow, Russian Federation

**Keywords:** chlorin, maleimide, porphyrin, spectral characteristics, synthesis

## Abstract

Maleimide-containing fluorinated porphyrins and chlorins were prepared based on the reaction of Zn(II) or Ni(II) complexes of 5,10,15,20-tetrakis(4-amino-2,3,5,6-tetrafluorophenyl)porphyrin and chlorin with maleic anhydride. Porphyrin maleimide derivatives were also prepared by the reaction of 5,10,15,20-tetrakis(4-azido-2,3,5,6-tetrafluorophenyl)porphyrinato Zn(II) or Ni(II) with *N*-propargylmaleimide via the CuAAC click reaction to afford fluorinated porphyrin–triazole–maleimide conjugates. New maleimide derivatives were isolated in reasonable yields and identified by UV–vis, ^1^H NMR, ^19^F NMR spectroscopy and mass-spectrometry.

## Introduction

Porphyrins belong to a broad class of the natural and synthetic macroheterocyclic compounds with unique properties. They play an important role in photosynthesis [1], catalysis [2–3], nonlinear optics [4–5], polymer synthesis [6] and energy conversions [7]. Porphyrins have been extensively studied as potential photosensitizers in a photodynamic therapy (PDT) [8–9], a promising treatment modality for several cancer and infectious diseases. In PDT, light, O_2_, and a photosensitizing drug are combined to produce a selective therapeutic effect via the generation of active oxygen forms (^1^O_2_, HO^−^, НО_2_^−^˙and O_2_^−^˙) upon excitation with monochromatic light which causes the death of the tumor [10–14]. Some other important features, that photosensitizers should have for such applications are their photo and thermal stability, and an ability to selectively accumulate in the target tissue, the absence of toxicity, toxic byproducts and mutagenic effects, and an opportunity for medical administration. An additional advantage of porphyrins is the possibility of functionalization of the macrocycle periphery with various substituents and thus to affect the photophysical, photochemical and tumor-specific properties of the porphyrin system. Such an approach provides a platform to new photosensitizers with optimized characteristics useful for biomedical applications including PDT. Currently a number of investigations directed for the preparation of tumor-targeted photosensitizers have been explored aiming to improve their tumor-specificity [15–16].

To continue our ongoing efforts on the preparation of porphyrin-based photosensitizers [17–20], we present herein the synthesis of maleimide-subtituted porphyrins and chlorins based on 5,10,15,20-tetrakis(pentafluorophenyl)porphyrin (**1**) as the starting compound. The presence of fluorine atoms on the four phenyl rings at the *meso*-positions of the porphyrin structure can make a strong influence on the hydrophobic interactions and lipophilicity, metabolic stability, thus modulating the biological efficiency of the photosensitizing agents [21–22]. At the same time fluorinated porphyrins are well known for their photostability and efficiency in generating long-lived triplet excited states through intersystem crossing (ISC) with minimal energy loss from excited states, and are widely used to generate singlet oxygen for PDT applications [9,23]. We intend here to combine within one molecule the structural specificity of a *meso*-fuorinated porphyrin/chlorin macrocycle and maleimide units to develop novel multifunctional compounds with improved properties for various applications. Maleimides are considered as a biologically important scaffold that possess almost all types of biological activities including antibacterial and antifungal activity [24], anticancer activity [25], cox-2 inhibitor and anti-inflammatory, antidiabetic activity [26] and photodynamic activity [27]. Attaching of the maleimide group with its rich biological activity to the tetrapyrrole macrocycles with their unique photophysical properties may result in new conjugates with improved chemical, biological and anticancer characteristics [28]. Moreover, maleimide is a stable functionality that rapidly and covalently conjugates thiol groups of cysteine residues in proteins or peptides by the thio-Michael addition to the double bond of the maleimide to form a corresponding succinimidyl thioether. Conjugation of the cysteine sulfhydryl group with maleimide moieties allows us to prepare the bioconjugates selectively, covalently in high yields with no requirement for prior activation of reactants [29] and thus strengthen the association of a drug molecule with the cell surface. It is important to mention that Kitagishi and co-workers [30] showed that the maleimide-appended porphyrin/cyclodextrine complex was conjugated to a cystein residue of serum albumin via a Michael addition reaction. At the same time, it is well known that 5,10,15,20-tetrakis(pentafluorophenyl)porphyrin and its chlorin derivatives generate singlet oxygen by the light irradiation under atmospheric oxygen [31]. These tetrapyrrole macrocycles and their metal complexes are considered to be efficient precursors for design and selection of new PDT agents, since their reactivity toward various nucleophiles provides a simple, selective and general access to the functionalized derivatives [32–34]. Considering the promise of porphyrins and chlorins for the development of PDT therapeutics and dependence of their biological properties on the structure of peripheral substituents, we developed a simple synthetic approach for new maleimide derivatives of the fluorinated porphyrins and chlorins. We also believe the synthetic potential of maleimide units in these new conjugates allows versatile ways to obtain a series of new photosensitizers for medical applications.

## Results and Discussion

### Synthesis of maleimide-substituted porphyrins

**Scheme 1:**
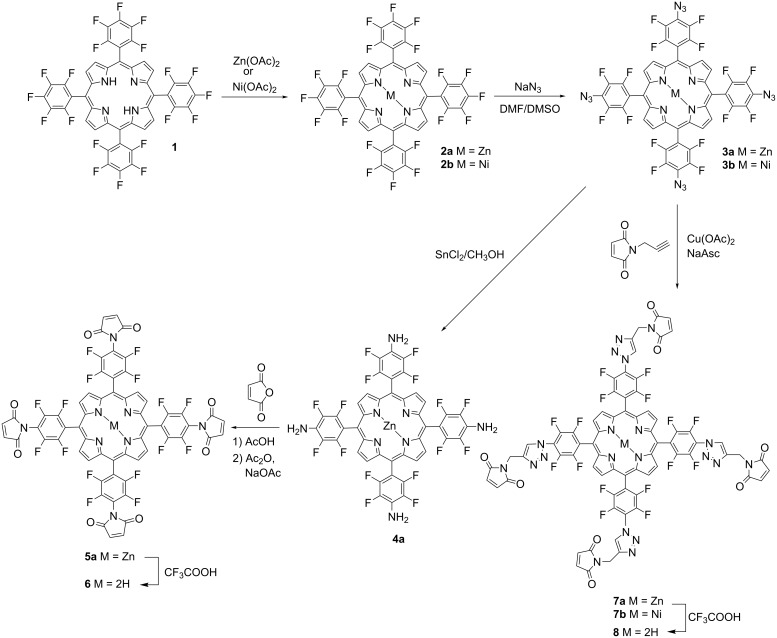
Synthesis of fluorinated maleimide-substituted porphyrins **5a**, **6**, **7a**, **7b**, and **8**.

In this work for the preparation of porphyrins functionalized with maleimde moieties commercially available 5,10,15,20-tetrakis(pentafluorophenyl)porphyrin (**1**) [35] was used as starting compound. The synthesis includes the metallation of the free base porphyrin **1** with an excess of zinc acetate or nickel acetate resulting in the corresponding porphyrin Zn(II) and Ni(II) complexes **2a** [36] and **2b** [37], respectively. Complexes **2a** and **2b** readily reacted with the excess of NaN_3_ in DMF/DMSO to afford porphyrins **3a** and **3b** in high yields via the selective substitution of fluorine atoms in the *para*-position of the pentafluorophenyl substituents with four azide functions. The subsequent reduction of *para*-tetraazidoporphyrinato Zn(II) **3a** with SnCl_2_ in MeOH [38] at ambient temperature smoothly provided the corresponding tetraamino-substituted porphyrin **4a** in 91% yield. (Scheme 1). The free amino groups of porphyrin **4a** are useful and reactive functionalities for further modifications. It was shown that the interaction of porphyrin **4a** with maleic anhydride by analogy with a well-known standard procedure [39–40] provided the target maleimide-substituted porphyrin **5a** in 42% yield, which contains four maleimide moieties in the structure. Treatment of porphyrin **5a** with trifluoroacetic acid in CHCl_3_ resulted in the removal of zinc from the coordination sphere of the porphyrin macrocycle and gave the free base maleimide porphyrin **6** in a quantitative yield. A successful formation of the fluorinated porphyrin azides **3a** and **3b** allowed their utility as intermediates in the further porphyrin core functionalisation especially with 1,2,3-triazole heterocycles via the copper-catalyzed azide–alkyne cycloaddition reaction (CuAAC) between alkynes and azides, developed independently by Sharpless [41] and Meldal [42]. In addition to the applications of triazoles as pharmacophores in the potential biologically active molecules, these heterocycles have also been used as linkers and for labeling biomolecules in chemical biology [43]. Moreover, this synthetic approach provides high yields, selectivity, mild reaction conditions and simple purification methods. It was demonstrated that the CuAAC reaction of porphyrins **3a** and **3b** with *N*-propargylmaleimide [44] was carried out successfully in CH_2_Cl_2_ and the fluorinated porphyrin–triazole–maleimide conjugates **7a** and **7b** were obtained in 54–58% yield. In these conjugates the tetrafluorophenyl units of the porphyrin macrocycle where separated from maleimides with 1,2,3-triazole spacer groups. Removal of zinc in porphyrin **7a** under the action of CF_3_COOH in CHCl_3_ resulted in porphyrin **8** in a quantitative yield (Scheme 1).

### Synthesis of maleimide-substituted chlorins

**Scheme 2:**
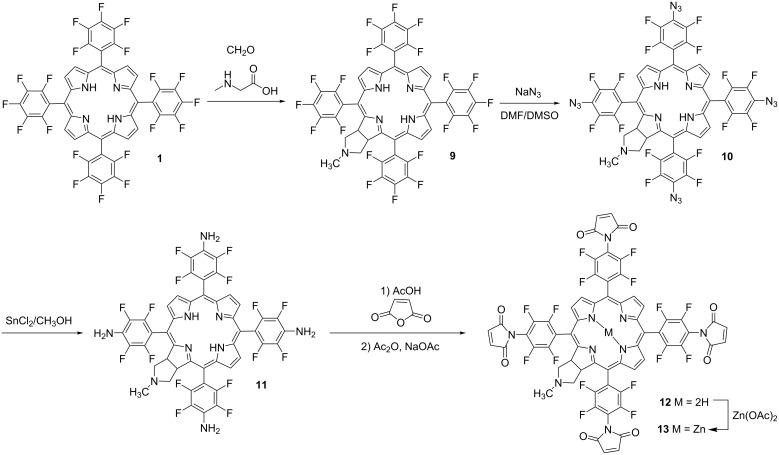
Synthesis of fluorinated maleimide-substituted chlorins **12**,**13**.

Following the developed procedure, maleimide-substituted chlorins were also prepared. Chlorins reveal a number of applications in the photobiological processes demonstrating suitable properties for PDT [45–47]. In this context the synthesis of new chlorins is one of the most important possibilities for the production of new efficient photosensitizers. The reason for this is that chlorins absorb light intensely at wavelengths where the human tissues are the most transparent [48]. Based upon our previous successful results with porphyrins we studied the possibility to obtain maleimide derivatives of 5,10,15,20-tetrakis(pentafluorophenyl)-17,18-*N*-methylpyrrolidine chlorin **9** [49] prepared by the reaction of porphyrin **1** with azomethine ylide, generated in situ from sarcosine and paraformaldehyde. The reaction of chlorin **9** with the excess of NaN_3_ in DMF/DMSO resulted in tetraazide chlorin **10** in 85% yield after the purification. The reduction of azide groups in chlorin **10** with SnCl_2_ in MeOH gave tetraamino-substituted chlorin **11** which was transformed into the corresponding maleimide **12** in 25% yield on the reaction with maleic anhydride according to the standard procedure. Chlorin **12** was metalated with Zn(OAc)_2_/CHCl_3_/MeOH giving chlorin **13** in 92% yield. (Scheme 2).

All prepared compounds have been structurally identified by ^1^H, ^19^F NMR, IR and UV–vis absorption spectra (see Supporting Information File 1).

## Conclusion

In this work, we developed a facile protocol for the preparation of new porphyrin and chlorin conjugates with maleimide entities which were synthesized in reasonable isolated yields and fully characterized with NMR spectroscopy and mass spectrometry. These novel porphyrins and chlorins are expected to be efficient photosensitizers for cancer treatment. The presence of four maleimide groups in these compounds may provide improved binding with proteins in living systems.

## Supporting Information

File 1 Experimental procedures, characterization data and copies of NMR and mass spectra of the synthesized compounds.
