# Supplementary material for: Fluorinated maleimide-substituted porphyrins and chlorins: synthesis and characterization
Source: Beilstein J Org Chem. 2019 Nov 13;15:2704–9. doi: 10.3762/bjoc.15.263 (PMC6880841; doi:10.3762/bjoc.15.263)
Supplement: File 1 — Experimental procedures, characterization data and copies of NMR and mass spectra of the synthesized compounds. [file Beilstein_J_Org_Chem-15-2704-s001.pdf]

BEILSTEIN JOURNAL OF ORGANIC CHEMISTRY

Supporting Information

for

Fluorinated maleimide-substituted porphyrins and chlorins: synthesis and characterization

Valentina A. Ol'shevskaya, Elena G. Kononova and Andrei V. Zaitsev

Beilstein J. Org. Chem. **2019,** *15,* 2704–2709. doi:10.3762/bjoc.15.263

Experimental procedures, characterization data and copies of NMR and mass spectra of the synthesized compounds

License and Terms: This is a supporting information file under the terms of the Creative Commons Attribution License (http://creativecommons.org/licenses/by/4.0). Please note that the reuse, redistribution and reproduction in particular requires that the authors and source are credited.
The license is subject to the *Beilstein Journal of Organic Chemistry* terms and conditions: (https://www.beilstein-journals.org/bjoc)

Experimental

General aspects. All reactions were performed in an atmosphere of dry argon. All solvents were dried according to the standard protocols. ^{1}H and ^{19}F NMR spectra were recorded on a Bruker Avance-400 spectrometer operating at 400.13 MHz for ^{1}H NMR and 376.5 MHz for ^{19}F NMR. Chemical shifts (δ) were referenced to the residual solvent peak ($CDCl_3$, ^{1}H: 7.26 ppm and $(CD_3)_2CO$, ^{1}H: 2.05 ppm) for ^{1}H, and external $CFCl_3$ for ^{19}F. IR spectra were recorded on a Bruker FTIR spectrometer Tensor 37 in KBr tablets. The UV–vis spectra were measured on a Carl Zeis Specord M 40 spectrophotometer in CH_2Cl_2 and acetone. The mass spectra were obtained using a VISION 2000 (MALDI) mass spectrometer; the most intense peaks are given for each compound. The identities of new compounds were verified by TLC on Sorbfil and Kieselgel 60 F254 (Merck) plates.

Merck silica gel L 0.040–0.080 mesh was used for column chromatography (elution with chloroform/hexane 2:1 for compounds **3a** and **3b;** chloroform/acetone 10:1 for compounds **9** and **10**; chloroform/acetone 5:2 for compounds **5a**, **7a**, **7b**, and **11;** chloroform/acetone 1:1 for compounds **4a**, **6**, **8**, **12** and **13**). Synthesis of compounds (**1**, **2a**, **2b**) has been reported earlier [1-3].

5,10,15,20-Tetrakis(4-azido-2,3,5,6-tetrafluorophenyl)porphyrinato zinc (II) (3a): Sodium azide (1.25 g, 19.26 mmol) was suspended in dry DMSO (15 mL) under an argon atmosphere and stirred for 15 min. To this suspension porphyrin **2a** (1.0 g, 0.963 mmol) in dry DMF (200 mL) was added at room temperature and then the reaction mixture was heated for 4 h at 65–70 °C under argon. Then the reaction mixture was allowed to reach rt, was poured into water (250 mL) and extracted with $CHCl_3$ (4 × 150 mL). The extracts were washed with water (2 × 250 mL), dried over Na_2SO_4, filtered and solvent was removed in vacuo. The residue was purified by column chromatography on silica gel, using a $CHCl_3$/hexane mixture (2:1) as an eluent, to give azido-substituted porphyrin **3a** (920 mg,

84.5%) as a red-violet powder. UV–vis (CH_2Cl_2): λ_{max}, nm (log ε) 422 (307.7), 550 (32.4). IR (KBr) ν_{max}, cm^{-1}: 2920 (porphyrin CH), 2123 (N_3). ^1H NMR (400.13 MHz, $CDCl_3$) δ: 9.10 (s, 8H, β-pyrrole). ^{19}F NMR (376.5 MHz, $CDCl_3$) δ: −151.9 (dd, *J* = 22.0, 8.2 Hz, 8F), −137.3 (dd, *J* = 22.0, 8.2 Hz, 8F). MS (MALDI): *m/z* [M^+] calcd. for $C_{44}H_8F_{16}N_{16}Zn$ 1128.015; found 1128.002.

5,10,15,20-Tetrakis(4-azido-2,3,5,6-tetrafluorophenyl)porphyrinato nickel (II) (3b) [4]**:** Sodium azide (1.26 g, 19.4 mmol) was suspended in dry DMSO (20 mL) under an argon atmosphere and stirred for 15 min. To this suspension porphyrin **2b (**1.0 g, 0.97 mmol) in dry DMF (200 mL) was added at room temperature and then the reaction mixture was heated for 6 h at 65–70 °C under argon. Then the reaction mixture (room temperature) was poured into water (250 mL) and extracted with $CHCl_3$ (4 × 150 mL).The extracts were washed with water (2 × 250 mL), dried over Na_2SO_4, filtered and solvent was removed in vacuo. The residue was purified by column chromatography on silica gel using a $CHCl_3$/ hexane mixture (2:1) as an eluent to give azido-substituted porphyrin **3b** (790 mg, 72.4%) as a dark purple powder. UV–vis (CH_2Cl_2): λ_{max}, nm (log ε) 418 (287.0), 552 (28.6). IR (KBr) ν_{max}, cm^{-1}: 2923 (porphyrin CH), 2125 (N_3). ^1H NMR (400.13 MHz, $CDCl_3$) δ: 9.08 (s, 8H, β-pyrrole). ^{19}F NMR (376.5 MHz, $CDCl_3$) δ – 151.9 (dd, *J* = 22.0, 8.3 Hz, 8F), –137.36 (dd, *J* = 22.0, 8.3 Hz, 8F). MS (MALDI): *m/z* [M^+] calcd. for $C_{44}H_8F_{16}N_{16}Ni$ 1122.022; found 1122.000.

5,10,15,20-Tetrakis(4-amino-2,3,5,6-tetrafluorophenyl) porphyrinato zinc (II) (4a): To a suspension of $SnCl_2$ (671 mg, 3.54 mmol) in dry MeOH (20 mL) azidoporphyrin (**3a**) (500 mg, 0.442 mmol) was added in MeOH (50 mL). The reaction mixture was stirred under argon for 1 h at room temperature. MeOH was removed in vacuo, the residue was purified by column chromatography on silica gel, using a $CHCl_3$/acetone (1:1) mixture as an eluent to afford amino-substituted porphyrin **4a** (415 mg, 91.4%) as a dark purple powder. UV–vis

(acetone): λ_{max}, nm (log ε) 420 (308.0), 551 (33.6). IR (KBr) ν_{max}, cm^{-1}: 2922 (porphyrin CH), 3430 (NH_2). ^{1}H NMR (400.13 MHz, $(CD_3)_2CO$) δ: 9.19 (s, 8H, β-pyrrole), 5.94 (s, 8H, NH_2). ^{19}F NMR (376.5 MHz, $(CD_3)_2CO$) δ: –164.51 (d, *J* = 16.5 Hz, 8F), –143.70 (d, *J* = 16.5 Hz, 8F). MS (MALDI): *m/z* [M^+] calcd. for $C_{44}H_{16}F_{16}N_8Zn$ 1024.053; found 1024.012.

5,10,15,20-Tetrakis(4-*N*-maleimido-2,3,5,6-tetrafluorophenyl)porphyrinato zinc (II) (5a): To a solution of porphyrin **4a** (300 mg, 0.292 mmol) in a mixture of glacial acetic acid (20 mL) and acetone (2 mL) maleic anhydride (572 mg, 5.84 mmol) was added and the reaction mixture was stirred in dark for 72 h under argon atmosphere at room temperature. After the removal of solvents in vacuo acetic anhydride (5 mL) and anhydrous NaOAc (479 mg, 5.84 mmol) were added to the residue and the mixture was heated at 50–60 °C for 24 h under argon. The solvent was removed under reduced pressure. The residue was adsorbed on silica gel and purified by column chromatography on silica gel ($CHCl_3$/acetone 5:2) to afford porphyrin **5a** (105 mg, 26.7%) as dark red solid. UV–vis (acetone): λ_{max}, nm (log ε) 419 (238.5), 550 (20.3). IR (KBr) ν_{max}, cm^{-1}: 1760 (C=O), 1611 (maleimide C=C). ^{1}H NMR (400.13 MHz, $(CD_3)_2CO$) δ: 9.35 (s, 8H, β-pyrrole), 7.46 (s, 8H, CH=CH). ^{19}F NMR (376.5 MHz, $(CD_3)_2CO$) δ: –145.8 (dd, *J* = 23.2, 10.7 Hz, 8F), –139.2 (dd, *J* = 23.2, 8.9 Hz, 8F). MS (MALDI): *m/z* [M^+] calcd. for $C_{60}H_{16}F_{16}N_8O_8Zn$ 1344.013; found 1344.000.

5,10,15,20-Tetrakis(4-*N*-maleimido-2,3,5,6-tetrafluorophenyl)porphyrin (6): To a solution of porphyrin **5a** (50 mg, 0.037 mmol) in $CHCl_3$ (10 mL) TFA (1.0 mL) was added and the mixture was stirred for 1 h at room temperature. The reaction mixture was poured into water (15 mL), the organic phase was separated and the solvent was removed under reduced pressure. Purification by column chromatography on silica gel ($CHCl_3$/acetone 1:1) afforded porphyrin **6** (47 mg, 98.6%) as dark red solid. UV–vis (acetone): λ_{max}, nm (log ε) 409 (301.5), 504 (27.4), 581 (12.7). IR (KBr) ν_{max}, cm^{-1}: 2920 (porphyrin CH), 1763

(C=O), 1613 (maleimide C=C). ^{1}H NMR (400.13 MHz, $(CD_3)_2CO$) δ: 9.36 (m, 8H, β-pyrrole), 7.46 (s, 8H, CH=CH), –2.66 (br.s, 2H, NH). ^{19}F NMR (376.5 MHz, $(CD_3)_2CO$) δ: −145.8 (dd, *J* = 23.2, 10.7 Hz, 8F), – 139.1 (dd, *J* = 23.2, 10.7 Hz, 8F). MS (MALDI): *m/z* [M^+] calcd. for $C_{60}H_{18}F_{16}N_8O_8$ 1282.099; found 1282.004.

5,10,15,20-Tetrakis[4-(4'-*N*-maleimidomethyl-1',2',3'-triazole-1'-yl)-2,3,5,6-tetrafluorophenyl]porphyrinato zinc (II) (7a): To a solution of porphyrin **3a** (100 mg, 0.0885 mmol) and N-propargylmaleimide [5] (72 mg, 0.531mmol) in CH_2Cl_2 (20 mL) the solution of $Cu(OAc)_2$ (3 mg/H_2O, 0.5 mL) and solution of sodium ascorbate (6 mg /H_2O, 0.5 mL) were added. The reaction mixture was boiled for 48 h with stirring and after this washed with water (3 × 30 mL). The solvent was removed under reduced pressure. The residue was adsorbed on silica gel and purified by column chromatography on silica gel ($CHCl_3$/acetone 5:2) to afford porphyrin **7a** (86 mg, 58.2%) as dark red solid. UV–vis (acetone): λ_{max}, nm (log ε) 418 (469.0), 550 (33.4). IR (KBr) ν_{max}, cm^{-1}: 2923 (porphyrin CH), 1770 (C=O), 1616 (maleimide C=C). ^{1}H NMR (400.13 MHz, $(CD_3)_2CO$) δ: 9.45 (s, 8H, β-pyrrole), 8.66 (s, 4H, triazole CH), 7.36 (s, 8H, CH=CH), 5.04 (s, 8H, CH_2). ^{19}F NMR (376.5 MHz, $(CD_3)_2CO$) δ: –149.7 (s, 8F), –138.7 (s, 8F). MS (MALDI): *m/z* [M^+] calcd. for $C_{72}H_{28}F_{16}N_{20}O_8Zn$ 1668.144; found 1668.038.

5,10,15,20-Tetrakis[4-(4'-*N*-maleimidomethyl-1',2',3'-triazole-1'-yl)-2,3,5,6-tetrafluorophenyl]porphyrinato nickel (II) (7b): To a solution of porphyrin **3b** (100 mg, 0.089 mmol) and *N*-propargylmaleimide [5] (96 mg, 0.712 mmol) in CH_2Cl_2 (20 mL) the solution of $Cu(OAc)_2$ (3 mg/H_2O, 0.5 mL) and a solution of sodium ascorbate (6 mg/H_2O, 0.5 mL) were added. The reaction mixture was boiled for 72 h with stirring and after this washed with water (3 × 30 mL). The solvent was removed under reduced pressure. The residue was adsorbed on silica gel and purified by column chromatography on silica gel ($CHCl_3$/acetone 5:2) to afford porphyrin **7b** (80 mg, 53.9%) as dark red solid. UV–vis

(acetone): λ_{max}, nm (log ε) 417 (426.0), 551 (31.2). IR (KBr) ν_{max}, cm^{-1}: 2923 (porphyrin CH), 1773 (C=O), 1620 (maleimide C=C). ^{1}H NMR (400.13 MHz, $(CD_3)_2CO$) δ: 9.43 (m, 8H, β-pyrrole), 8.62 (s, 4H, triazole CH), 7.35 (s, 8H, CH=CH), 5.03 (s, 8H, CH_2). ^{19}F NMR (376.5 MHz, $(CD_3)_2CO$) δ: –149.0 (s, 8F), –138.8 (s, 8F). MS (MALDI): *m/z* [M^+] calcd. for $C_{72}H_{28}F_{16}N_{20}O_8Ni$ 1662.150; found 1662.076.

5,10,15,20-Tetrakis[4-(4'-*N*-maleimidomethyl-1',2',3'-triazole-1'-yl)-2,3,5,6-tetrafluorophenyl]porphyrin (8): To a solution of porphyrin **7a** (40 mg, 0.024 mmol) in $CHCl_3$ (5 mL) TFA (0.5 mL) was added and the mixture was stirred for 1 h at room temperature. Then the reaction mixture was poured into water (15 mL), the organic phase was separated and the solvent was removed under reduced pressure. Purification by column chromatography on silica gel ($CHCl_3$/acetone 1:1) afforded porphyrin **8** (38 mg, 95.8%) as dark red solid. UV–vis (acetone): λ_{max}, nm (log ε) 411 (247.0), 506 (25.7), 543 (13.7), 584 (15.2), 634 (10.7). IR (KBr) ν_{max}, cm^{-1}: 2925 (porphyrin CH), 1775 (C=O), 1614 (maleimide C=C). ^{1}H NMR (400.13 MHz, $(CD_3)_2CO$) δ: 9.58 (m, 8H, β-pyrrole), 8.67 (s, 4H, triazole CH), 7.05 (s, 8H, CH=CH), 5.06 (s, 8H, CH_2), –2.82 (s, 2H, NH). ^{19}F NMR (376.5 MHz, $(CD_3)_2CO$) δ: –149.1 (m, 8F), –138.8 (m, 8F). MS (MALDI): *m/z* [M^+] calcd. for $C_{72}H_{30}F_{16}N_{20}O_8$ 1606.230; found 1606.131.

5,10,15,20-Tetrakis(pentafluorophenyl)-17,18-*N*-methylpyrrolidine chlorin (9) [6]**:** To a solution of porphyrin **1** in dry toluene (250 mL) formaldehyde (306 mg, 4.08 mmol) and sarcosine (363 mg, 4.08 mmol) were added and the mixture was boiled under argon for 4 h. Then another portions of formaldehyde (306 mg, 10.2 mmol) and sarcosine (363 mg, 4.08 mmol) were added and the resulted mixture was boiled for 4 hours. The solvent was removed under reduced pressure, the residue was purified by column chromatography on silica gel ($CHCl_3$/acetone 10:1) to afford chlorin **9** (590 mg, 56.1%) as dark green solid. UV–vis (CH_2Cl_2): λ_{max}, nm (log ε): 406 (305.4), 504 (34.5), 598 (16.7), 651 (83.4). IR (KBr)

ν_{max}, cm^{-1}: 2926 (chlorin CH). ^{1}H NMR (400.13 MHz, $CDCl_3$) δ: 8.77 (d, *J* = 4.8 Hz, 2H, β-pyrrole), 8.53 (s, 2H, β-pyrrole), 8.44 (d, *J* = 4.8 Hz, 2H, β-pyrrole), 5.30 (t, *J* = 4.8 Hz, 2H, CH), 3.18 (t, *J* = 7.8 Hz, 2H, CH_2), 2.59 (t, *J* = 6.2 Hz, 2H, CH_2), 2.25 (s, 3H, Me), –1.77(s, 2H, NH). ^{19}F NMR (376.5 MHz, $CDCl_3$) δ –161.5 (dd, *J* = 38.5, 22.0 Hz, 4F), –160.5 (t, *J* = 16.5 Hz, 2F), –160.1 (t, *J* = 16.5 Hz, 2F), –151.7 (t, *J* = 19.2 Hz, 2F), –151.4 (t, *J* = 22.0 Hz, 2F), –137.3 (d, *J* = 22.0 Hz, 2F), –136.9 (t, *J* = 22.0 Hz, 4F), –135.4 (d, *J* = 24.8 Hz, 2F). MS (MALDI): *m/z* [M^+] calcd. for $C_{47}H_{17}F_{20}N_5$ 1031.116; found 1031.004.

5,10,15,20-Tetrakis(4-azido-2,3,5,6-tetrafluorophenyl)-17,18-*N*-methylpyrrolidine chlorin (10): Sodium azide (629 mg, 9.68 mmol) was suspended in dry DMSO (10 mL) under the argon atmosphere and stirred for 15 min. To this suspension chlorin **9** (500 mg, 0.484 mmol) in dry DMF (100 mL) was added and the reaction mixture was heated for 6 h at 65–70 °C under argon. Then the reaction mixture was allowed to reach room temperature, poured into water (150 mL) and extracted with $CHCl_3$ (4 × 100 mL). The extracts were washed with water (2 × 150 mL), dried over Na_2SO_4, filtered and solvent was removed under reduced pressure. The residue was purified by column chromatography on silica gel ($CHCl_3$/acetone 10:1) to afford chlorin **10** (465 mg, 85.6%) as dark green solid. UV–vis (CH_2Cl_2): λ_{max}, nm (log ε): 412 (340.0), 506 (43.6), 601(6.4), 654 (86.9). IR (KBr) ν_{max}, cm^{-1}: 2924 (chlorin CH), 2125 (N_3). ^{1}H NMR (400.13 MHz, $CDCl_3$) δ: 8.77 (s, 2H, β-pyrrole), 8.55 (d, *J* = 4.4 Hz, 2H, β-pyrrole), 8.45 (s, 2H, β-pyrrole), 5.30 (s, 2H, CH), 3.20 (s, 2H, CH_2), 2.56 (s, 2H, CH_2), 2.25 (s, 3H, Me), –1.78(s, 2H, NH). ^{19}F NMR (376.5 MHz, $CDCl_3$) δ –151.8 (dq, *J* = 22.0, 11.0 Hz, 4F), –150.9 (q, *J* = 11.0 Hz, 2F), –150.5 (q, *J* = 11 Hz, 2F), –137.9 (dd, *J* = 22.0, 8.2 Hz, 2F), –137.4 (t, *J* = 19.2 Hz, 4F), –135.9 (d, *J* = 13.7 Hz, 2F). MS (MALDI): *m/z* [M^+] calcd. for $C_{47}H_{17}F_{16}N_{17}$ 1123.160; found 1123.005.

5,10,15,20-Tetrakis(4-amino-2,3,5,6-tetrafluorophenyl)-17,18-*N*-methylpyrrolidine chlorin (11): $SnCl_2$ (540 mg, 2.85 mmol) was suspended in dry MeOH (20 mL) for 20 min. Then to this mixture azido chlorin (**10**) (400 mg, 0.356 mmol) was added in MeOH (50 mL). The reaction mixture was stirred under argon for 1 h at room temperature. MeOH was removed under reduced pressure, the residue was purified by column chromatography on silica gel ($CHCl_3$/acetone 5:2) to afford amino chlorin **11** (342 mg, 94.1%) as a dark green solid. UV–vis (acetone): λ_{max}, nm (log ε) 411 (334.2), 505 (47.2), 599(7.8), 653 (82.3). IR (KBr) ν_{max}, cm^{-1}: 2924 (chlorin CH), 3435 (NH_2). ^1H NMR (400.13 MHz, $(CD_3)_2CO$) δ: 9.17 (m, 2H, β-pyrrole), 8.82 (m, 4H, β-pyrrole), 5.97 (br.s, 8H, NH_2), 4.61 (br.s, 2H, CH), 3.22 (br.s, 2H, CH_2), 2.62 (s, 2H, CH_2), 2.16 (s, 3H, Me), –1.76 (br.s, 2H, NH). ^{19}F NMR (376.5 MHz, $(CD_3)_2CO$) δ: –147.5 (m, 2F), –146.5 (m, 4F), –145.9 (m, 2F), –141.0 (m, 2F), –140.7 (m, 4F), –139.2 (m, 2F). MS (MALDI): *m/z* [M^+] calcd. for $C_{47}H_{25}F_{16}N_9$ 1019.198; found 1019.037.

5,10,15,20-Tetrakis(4-*N*-maleimido-2,3,5,6-tetrafluorophenyl)-17,18-*N*-methylpyrrolidine chlorin (12). To a solution of chlorin **11** (300 mg, 0.294 mmol) in a mixture of glacial acetic acid (20 mL) maleic anhydride (576 mg, 5.88 mmol) was added and the reaction mixture was stirred in dark for 96 h under argon at room temperature. After the removal of solvent under reduced pressure acetic anhydride (5 mL) and anhydrous NaOAc (482 mg, 5.88 mmol) were added to the residue and the reaction mixture was heated at 50-60 ^0C for 24 h under argon in the dark. The solvent was removed under reduced pressure, the residue was purified by column chromatography on silica gel ($CHCl_3$/acetone 1:1) to afford chlorin **12** (87 mg, 22.1%) as dark green solid. UV-vis (acetone): λ_{max}, nm (log ε) 406 (253.4), 504 (31.0), 598 (14.5), 651 (65.0). IR (KBr) ν_{max}, cm^{-1}: 2924 (chlorin CH), 1765 (C=O), 1615 (maleimide C=C). ^1H NMR (400.13 MHz, $(CD_3)_2CO$) δ: 9.24 (m, 2H, β-pyrrole), 8.85 (m, 4H, β-pyrrole), 6.84 (dd, *J* = 12.1, 3.8 Hz,

4H, CH=CH), 6.55 (d, *J* = 12.1 Hz, 4H, CH=CH), 5.48 (br.s, 2H, CH), 3.21 (br.s, 2H, CH_2), 2.73 (br.s, 2H, CH_2), 2.37 (s, 3H, Me), –1.66 (br.s, 2H, NH). ^{19}F NMR (376.5 MHz, $(CD_3)_2CO$) δ: –146.2 (m, 2F), –145.2 (m, 4F), –144.8 (m, 2F), –141.6 (m, 4F), –139.7 (m, 2F), –139.3 (m, 2F). MS (MALDI): *m/z* [M^+] calcd. for $C_{63}H_{25}F_{16}N_9O_8$ 1339.157; found 1339.014.

Zinc complex of 5,10,15,20-Tetrakis(4-*N*-maleimido-2,3,5,6-tetrafluorophenyl)-17,18-*N*-methylpyrrolidine chlorin (13). Chlorin **12** (35 mg, 0.026 mmol) and $Zn(OAc)_2{\cdot}2H_2O$ (57 mg. 0.26 mmol) were dissolved in a mixture of $CHCl_3$ (10 mL) and MeOH (10 mL) and boiled for 4 h under argon in the dark. After the removal of the solvent in vacuo the residue was purified by column chromatography on silica gel ($CHCl_3$/acetone 1:1) to afford chlorin **13** (34 mg, 92.3%) as dark green solid. UV–vis (acetone): λ_{max}, nm (log ε) 409 (267.4), 505 (32.7), 649 (67.2). IR (KBr) ν_{max}, cm^{-1}: 2926 (chlorin CH), 1770 (C=O), 1614 (maleimide C=C). ^{1}H NMR (400.13 MHz, $(CD_3)_2CO$) δ: 9.25 (m, 6H, β-pyrrole), 6.94 (d, *J* = 12.4 Hz, 4H, CH=CH), 6.61 (d, *J* = 12.4 Hz, 4H, CH=CH), 5.42 (br.s, 2H, CH), 3.20 (br.s, 2H, CH_2), 2.69 (br.s, 2H, CH_2), 2.38 (s, 3H, Me). ^{19}F NMR (376.5 MHz, $(CD_3)_2CO$) δ: –147.5 (d, *J* = 13.7 Hz, 2F), –146.5 (d, *J* = 13.7 Hz, 4F), –145.9 (q, *J* = 11.0 Hz, 2F), –141.3 (d, *J* = 13.7 Hz, 2F), –140.7 (d, *J* = 13.7 Hz, 4F), –139.2 (dd, J = 22.0, 8.2 Hz, 2F). MS (MALDI): *m/z* [M^+] calcd. for $C_{63}H_{23}F_{16}N_9O_8Zn$ 1401.071; found 1401.003.

Literature

1. Banfi, S.; Caruso, E.; Caprioli, S.; Mazzagatti, L.; Canti, G.; Ravizza, R.; Gariboldi, M.; Monti, E. *Bioorg. Med. Chem.* **2004**, *12*, 4853–4860. doi: 10.1016/j.bmc.2004.07.011
2. Das, S. K.; Song, B.; Mahler, A.; Nesterov, V.N.; Wilson, A.K.; Ito, O.; D'Souza, F. *J. Phys. Chem.* **2014**, *118*, 3994–4006. doi: 10.1021/jp4118166

3. Peters, M.K.; Herges, R. *Inorg. Chem.* **2018,** *57*, 3177–3182. doi: 10.1021/acs.inorgchem.7b03164
4. Brenner, W.; Ronson, T.K.; Nitschke, J.R. *J. Am. Chem. Soc.* **2017**, *139*, 75-78. doi: 10.1021/jacs.6b11523
5. Yan, J.; Wang, R.; Pan, D.; Yang, R.; Xu, Y.; Wanga, L.; Yang, M. *Polym. Chem.* **2016**, *40***,** 6241-6249. doi: 10.1039/C6PY01344A
6. Jimenez-Oses, G.; Garcıa, J.I.; Silva, A.M.G.; Santos, A.R.N.; Tome, A.C.; Neves, M.G.P.M.S.; Cavaleiro, J.A.S. *Tetrahedron* **2008**, *64*, 7937–7943. doi:10.1016/j.tet.2008.06.018

NMR spectra

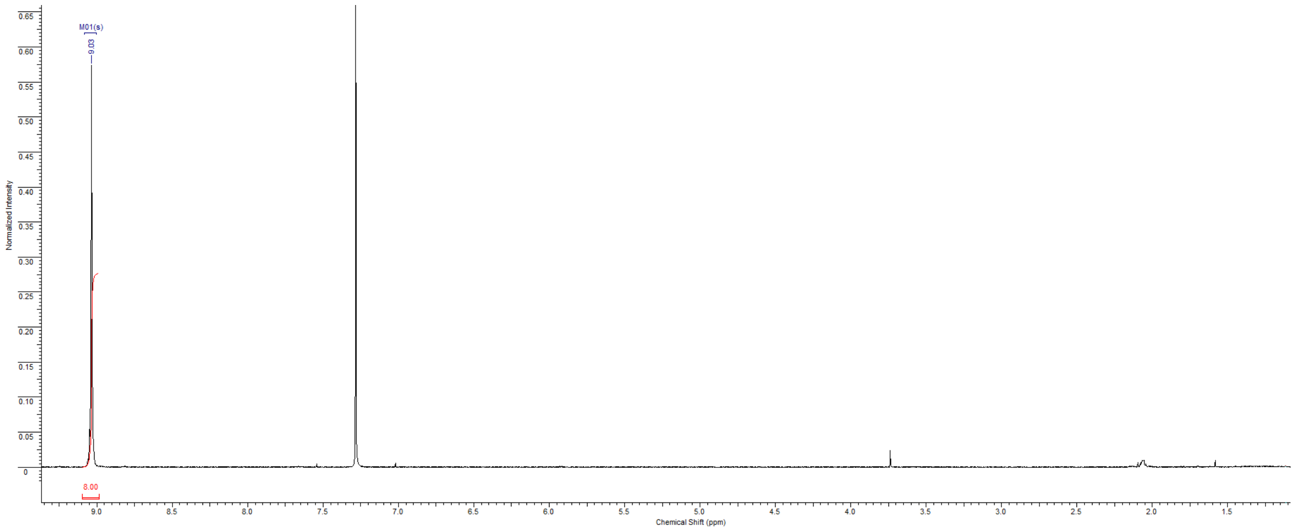

Figure S1 – ^{1}H NMR (400 MHz, $CDCl_3$) spectrum of compound **3a**.

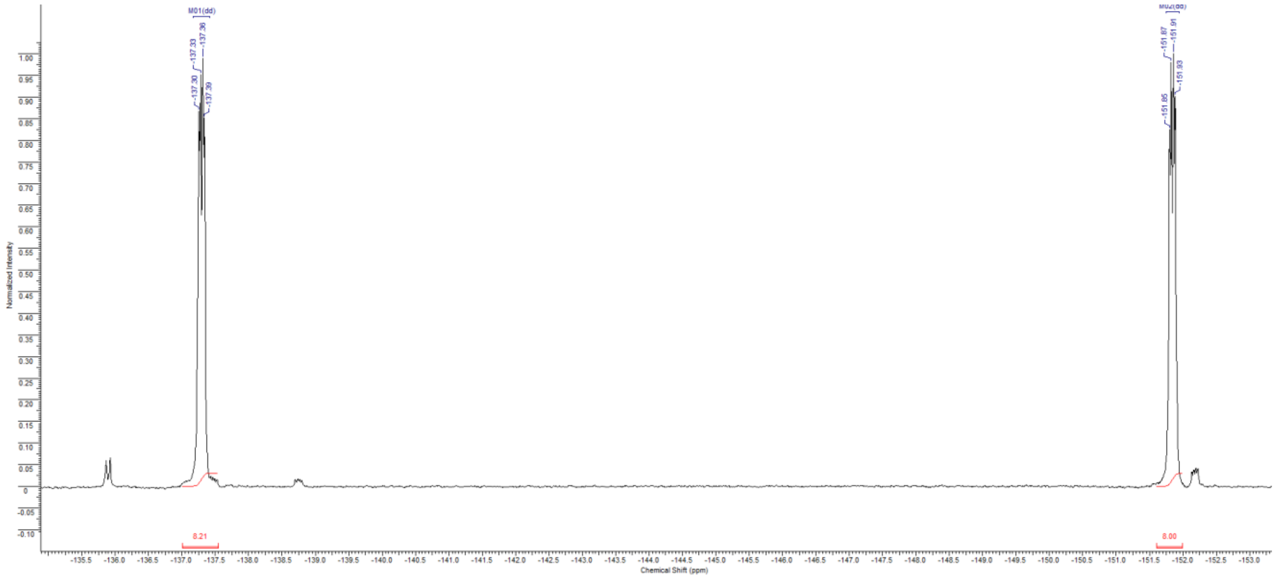

Figure S2 – ^{19}F NMR (376 MHz, $CDCl_3$) spectrum of compound **3a**.

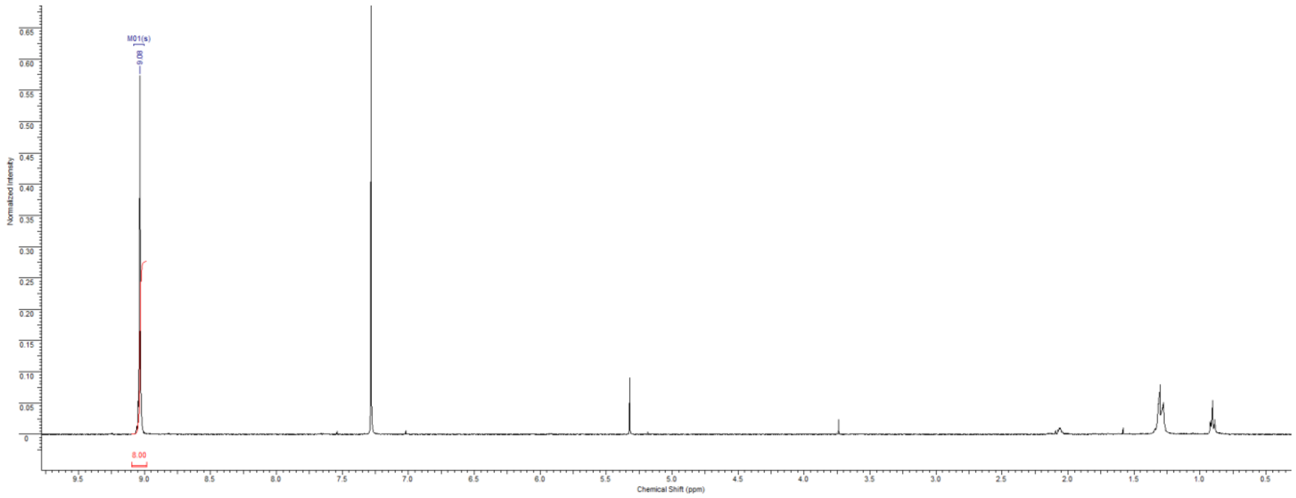

Figure S3 – ^{1}H NMR (400 MHz, $CDCl_3$) spectrum of compound **3b**.

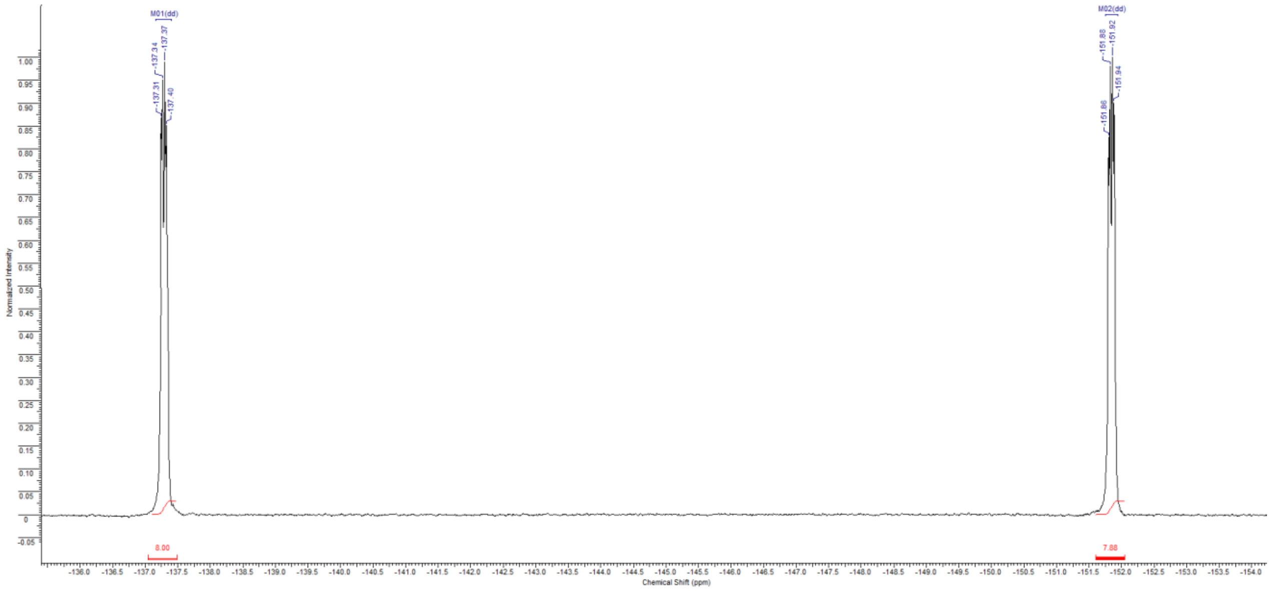

Figure S4 – ^{19}F NMR (376 MHz, $CDCl_3$) spectrum of compound **3b**.

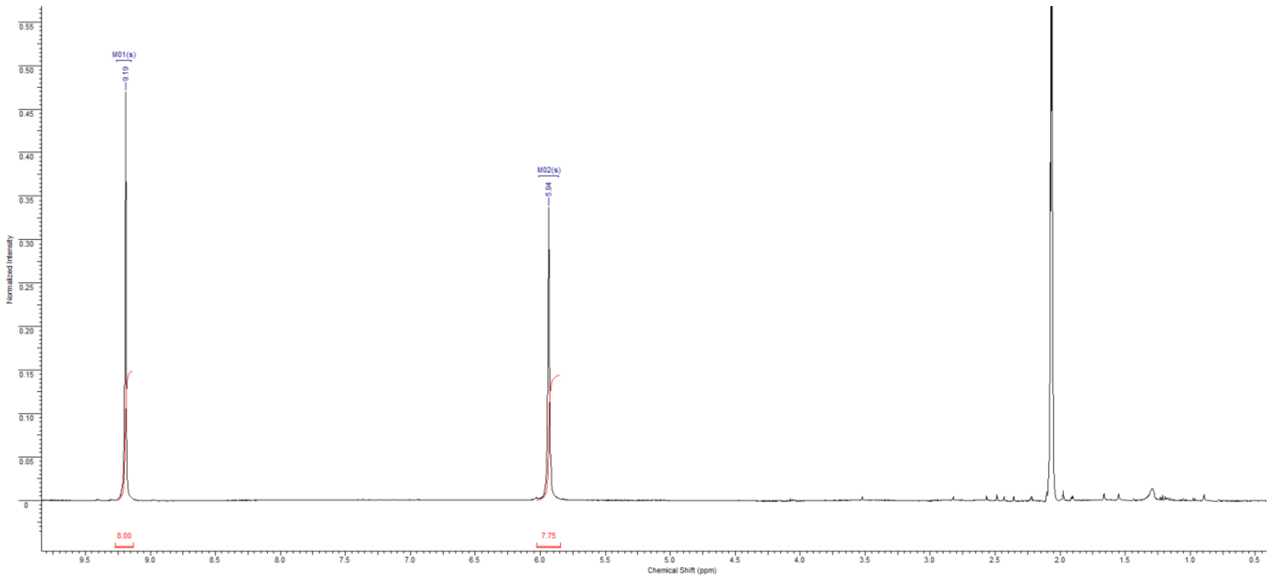

Figure S5 – ^{1}H NMR (400 MHz, $(CD_3)_2CO$) spectrum of compound **4a**.

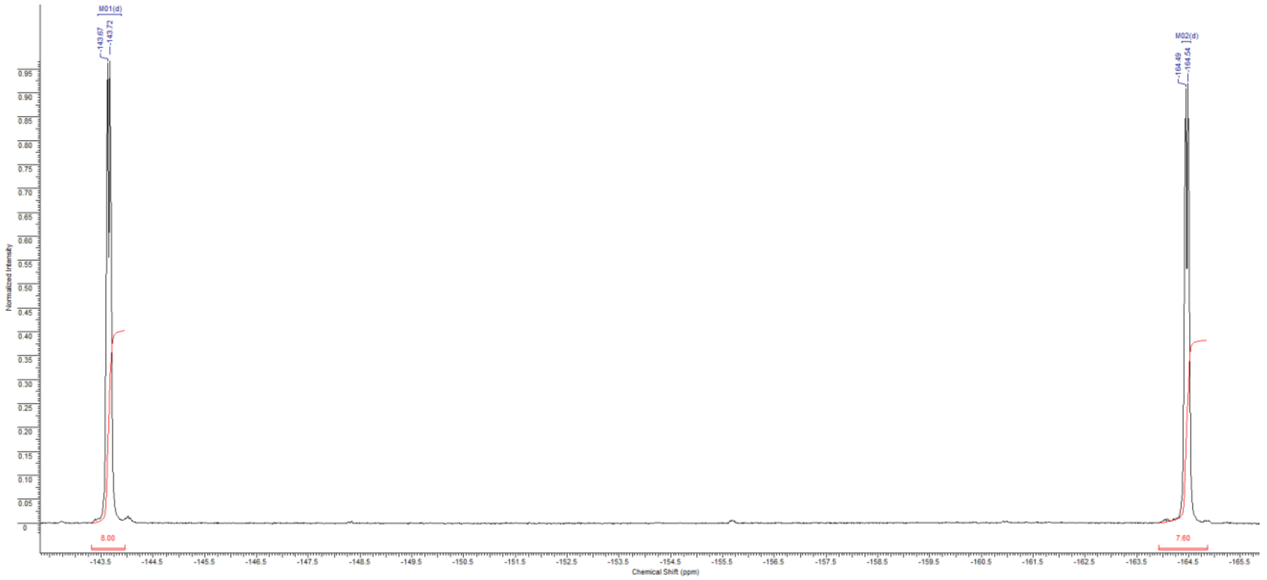

Figure S6 – ^{19}F NMR (376 MHz, $(CD_3)_2CO$) spectrum of compound **4a**.

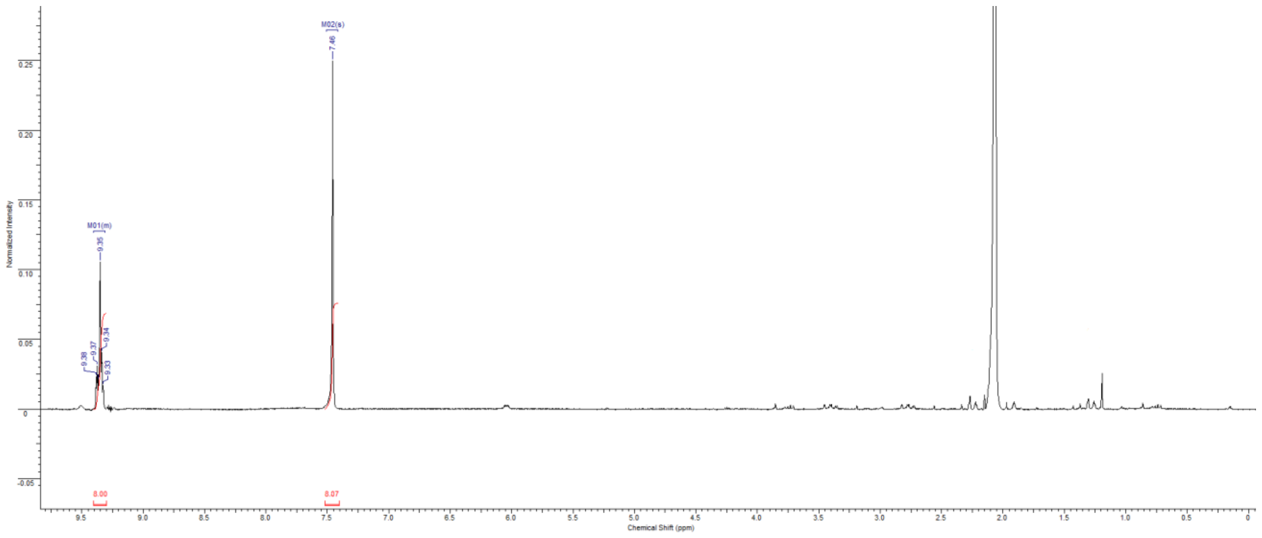

Figure S7 – ^{1}H NMR (400 MHz, $(CD_3)_2CO$) spectrum of compound **5a**.

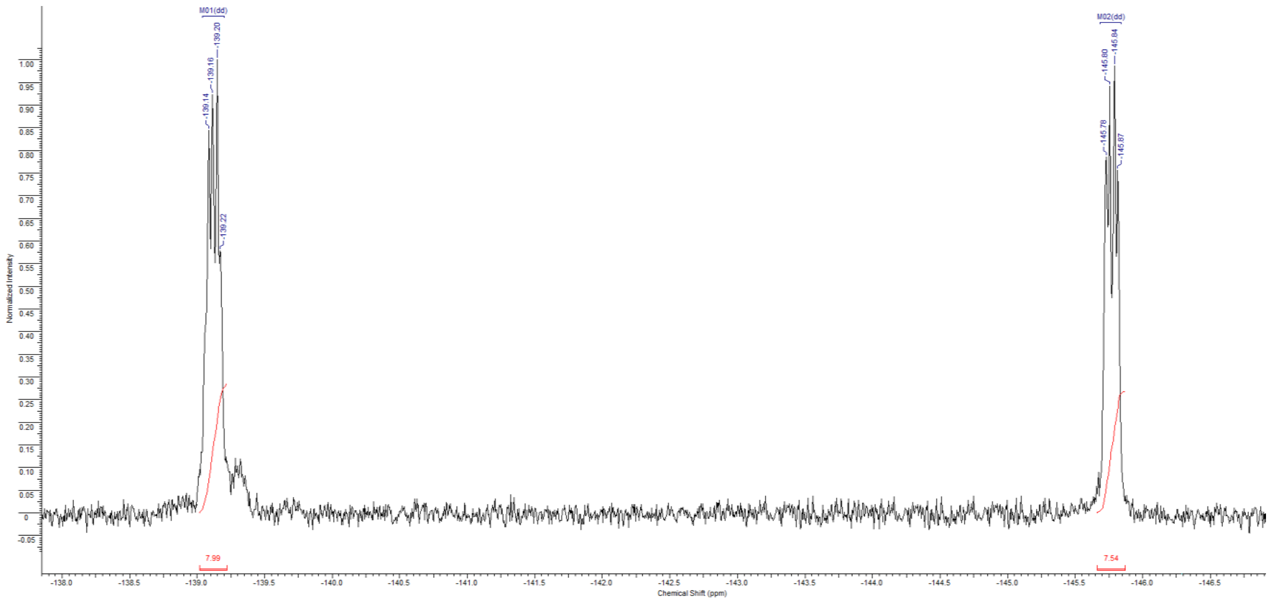

Figure S8 – ^{19}F NMR (376 MHz, $(CD_3)_2CO$) spectrum of compound **5a**.

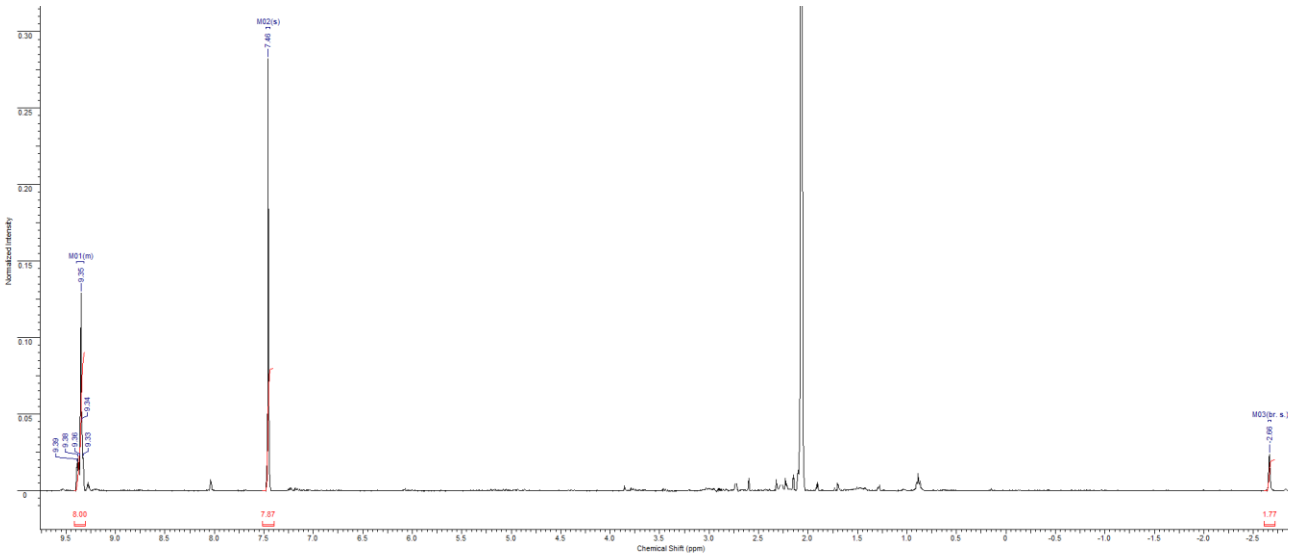

Figure S9 – ^{1}H NMR (400 MHz, $(CD_3)_2CO$) spectrum of compound **6**.

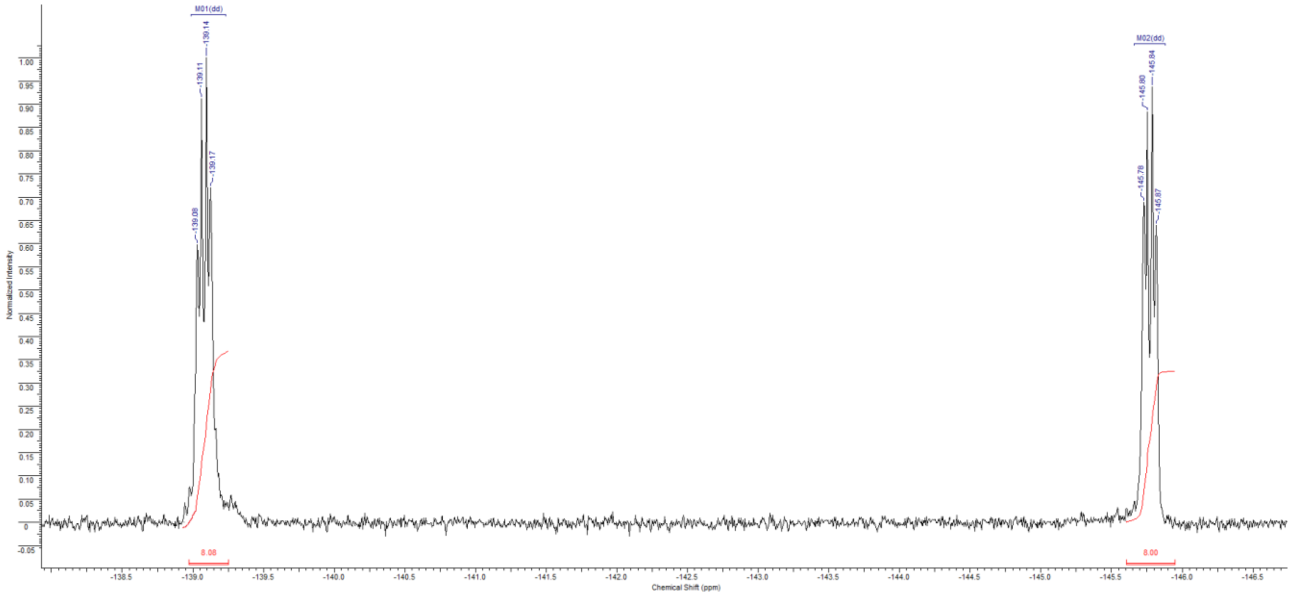

Figure S10 – ^{19}F NMR (376 MHz, $(CD_3)_2CO$) spectrum of compound **6**.

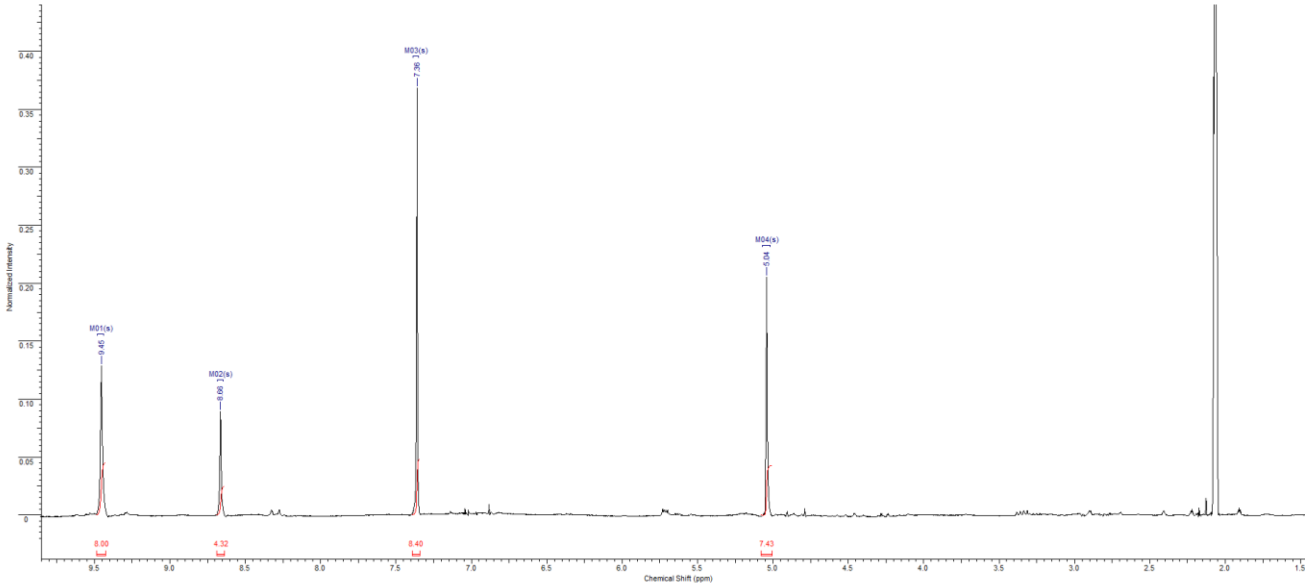

Figure S11 – ^{1}H NMR (400 MHz, $(CD_3)_2CO$) spectrum of compound **7a**.

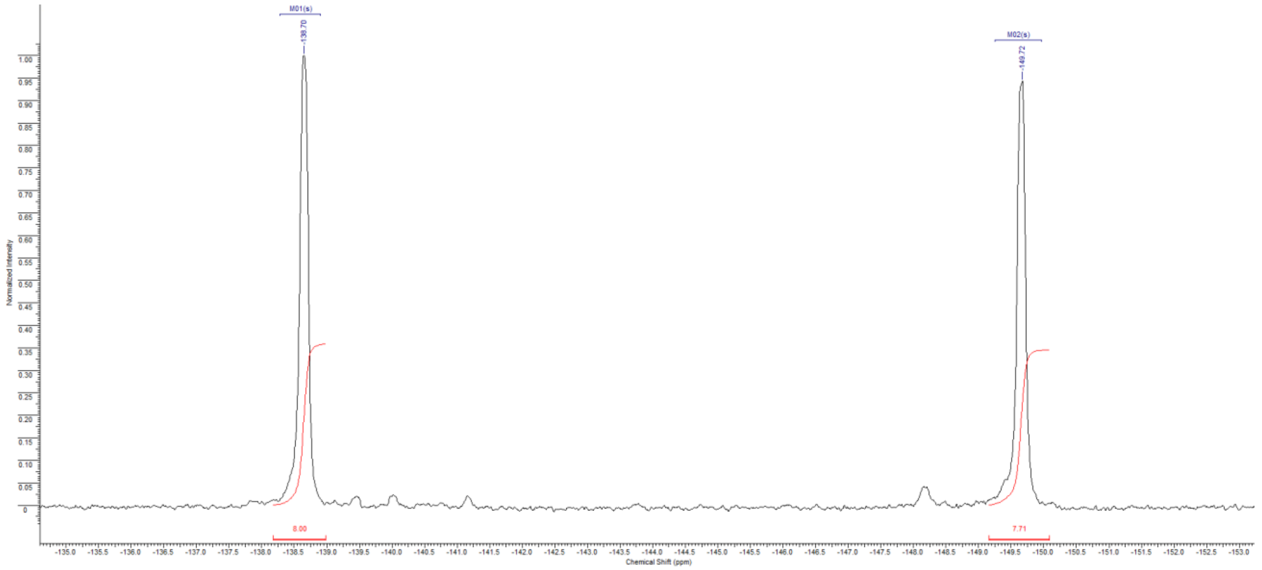

Figure S12 – ^{19}F NMR (376 MHz, $(CD_3)_2CO$) spectrum of compound **7a**.

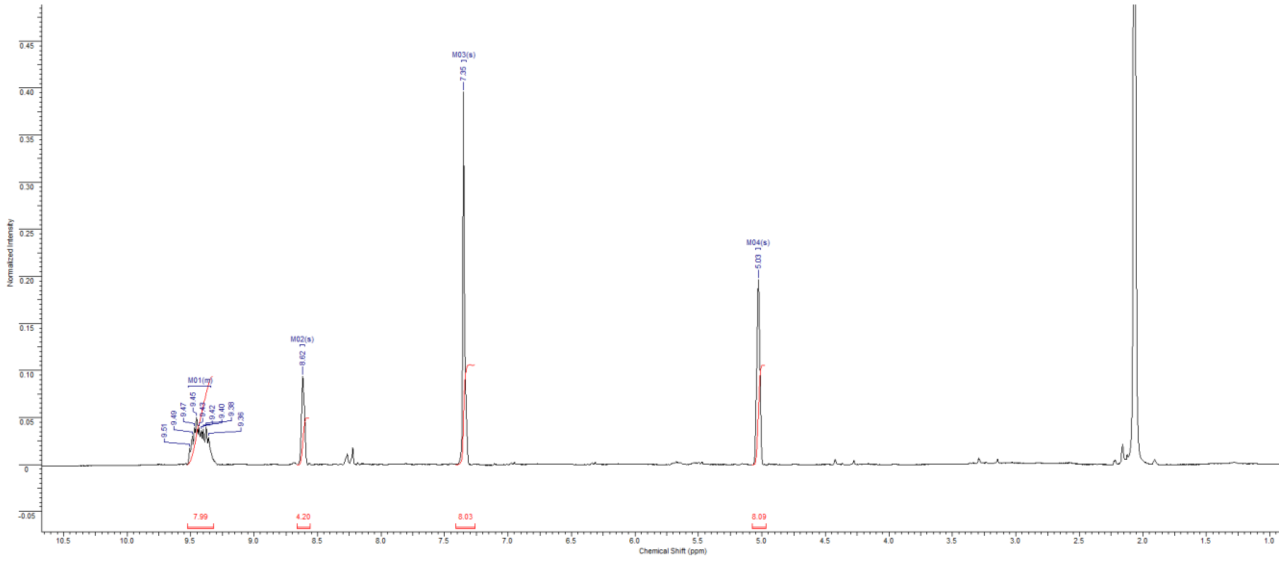

Figure S13 – ^{1}H NMR (400 MHz, $(CD_3)_2CO$) spectrum of compound **7b**.

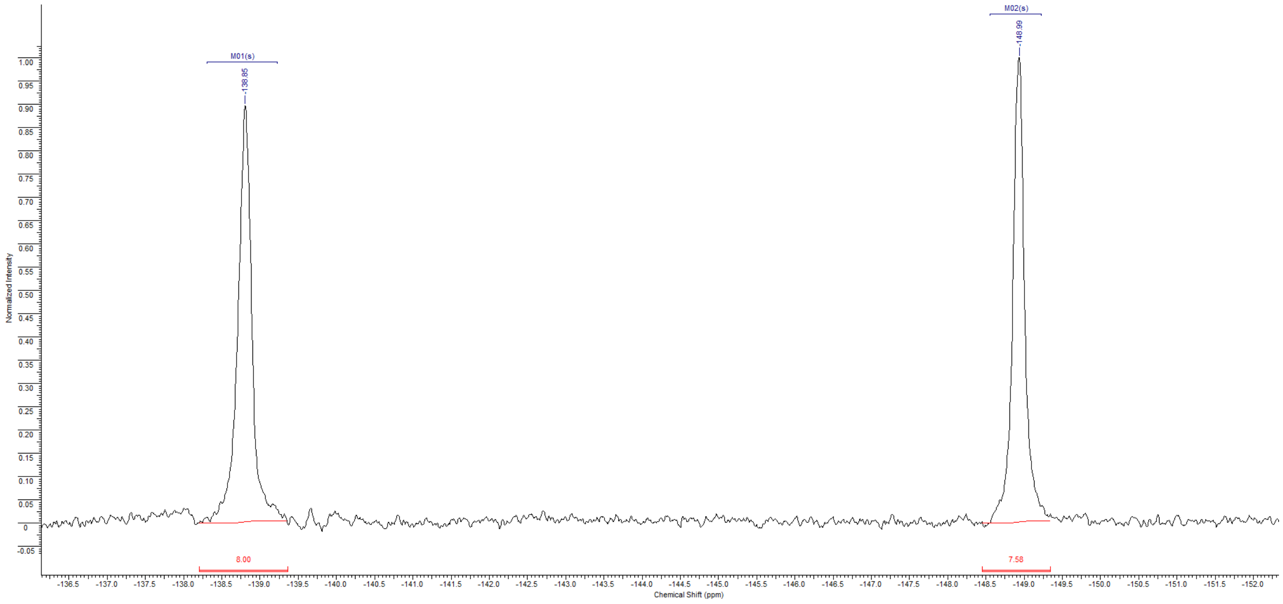

Figure S14 – ^{19}F NMR (376 MHz, $(CD_3)_2CO$) spectrum of compound **7b**.

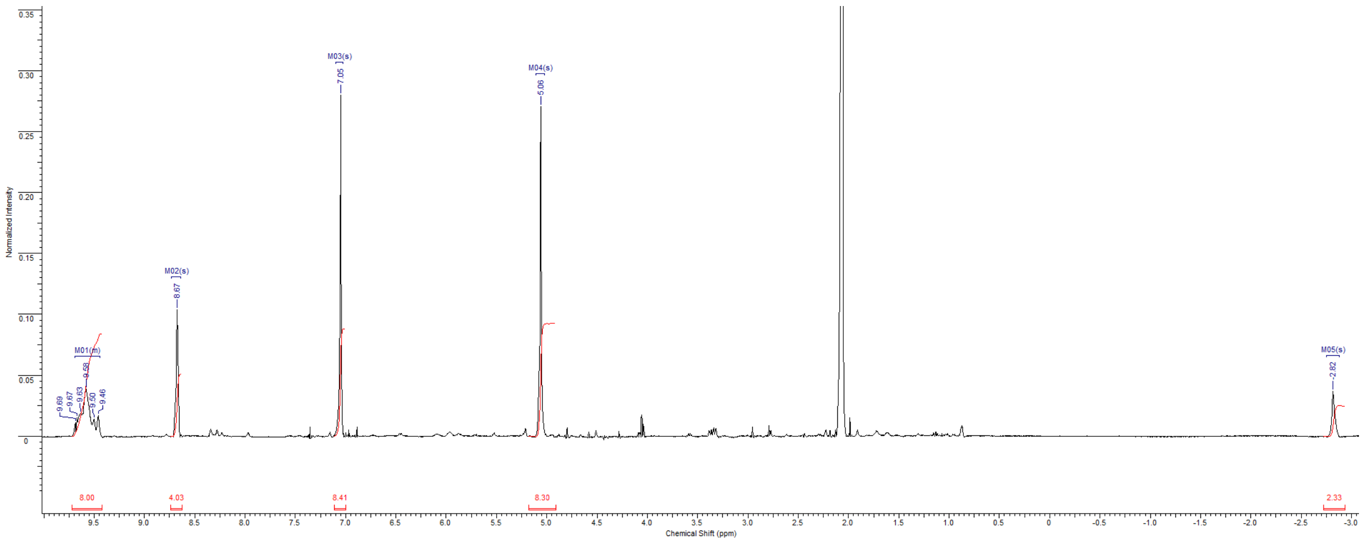

Figure S15 – ^{1}H NMR (400 MHz, $(CD_3)_2CO$) spectrum of compound **8**.

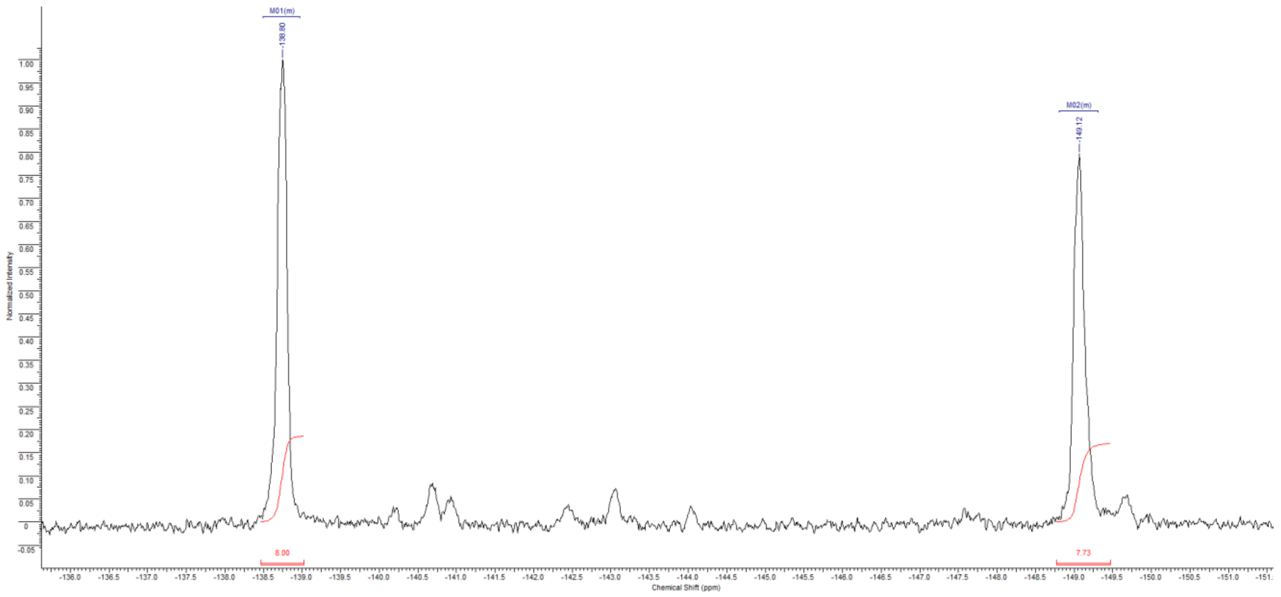

Figure S16 – ^{19}F NMR (376 MHz, $(CD_3)_2CO$) spectrum of compound **8**.

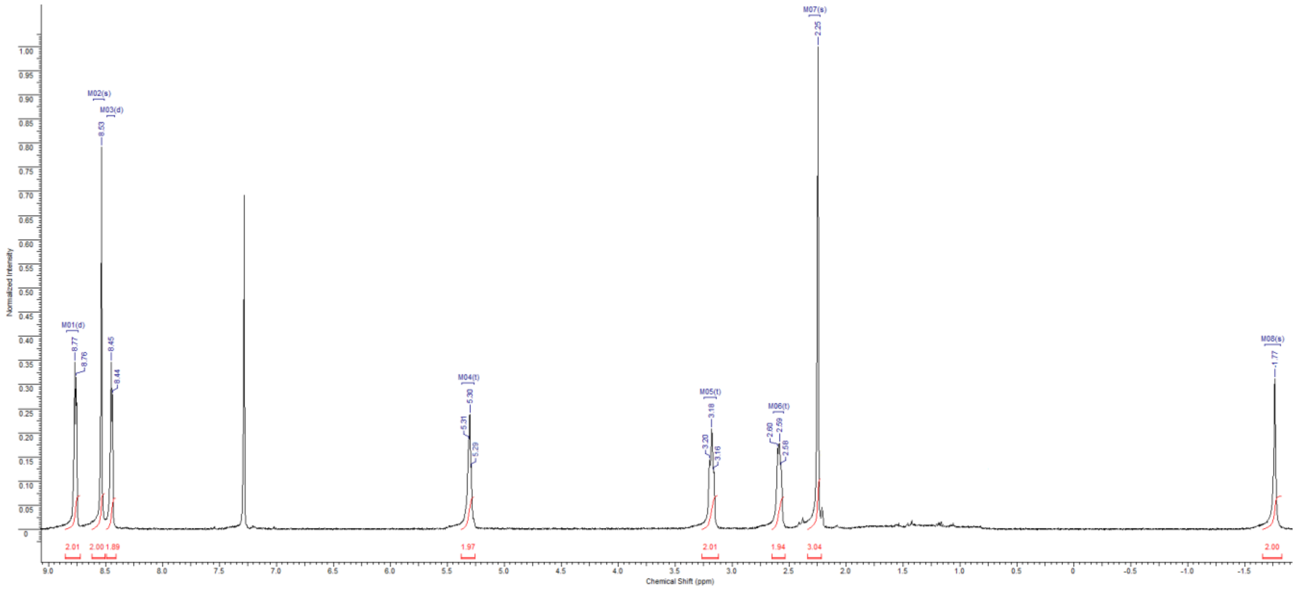

Figure S17 – ^{1}H NMR (400 MHz, $CDCl_3$) spectrum of compound **9**.

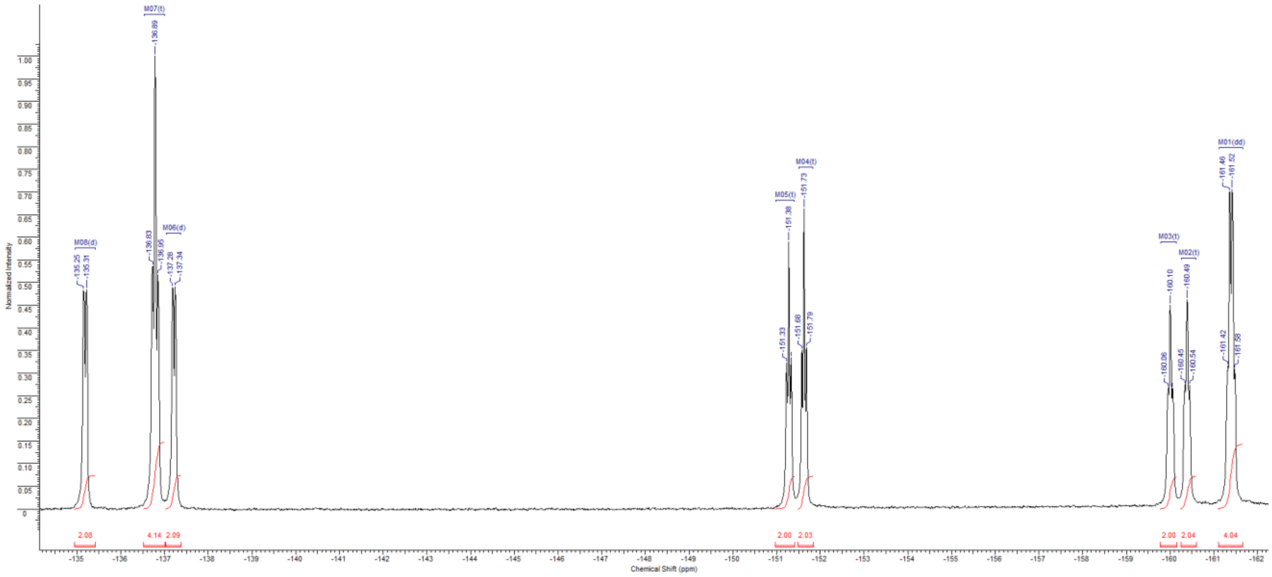

Figure S18 – ^{19}F NMR (376 MHz, $CDCl_3$) spectrum of compound **9**.

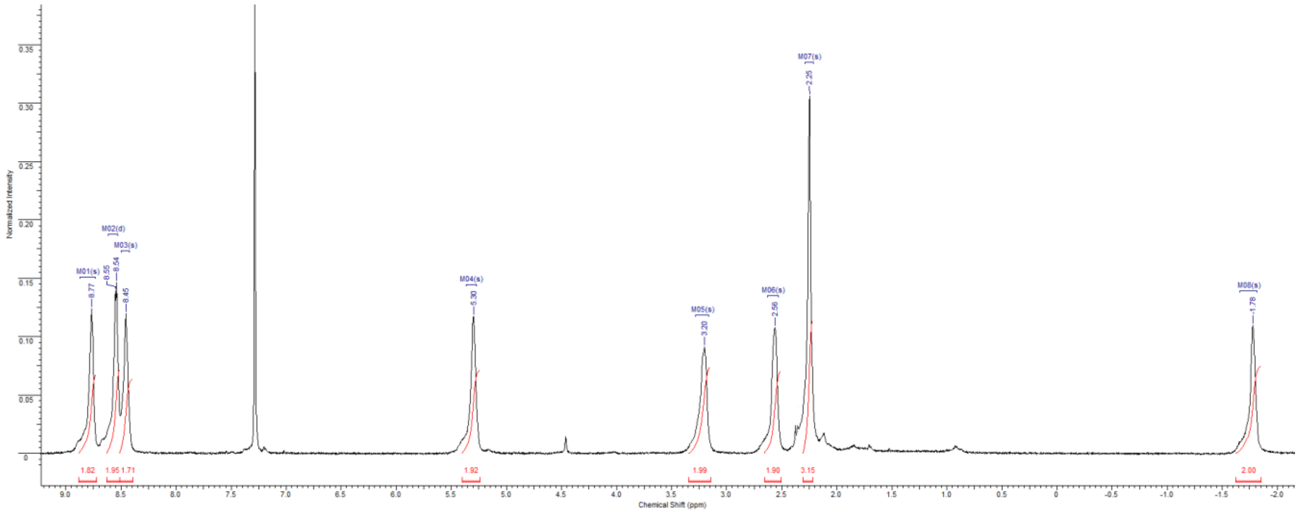

Figure S19 – ^{1}H NMR (400 MHz, $CDCl_3$) spectrum of compound **10**.

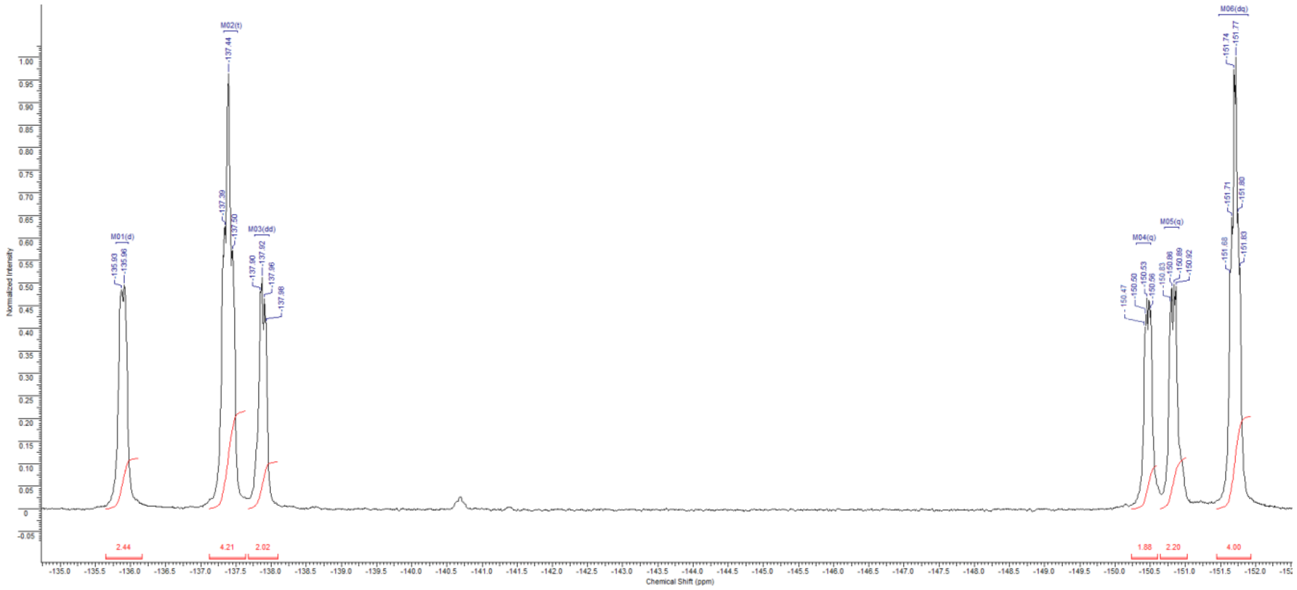

Figure S20 – ^{19}F NMR (376 MHz, $CDCl_3$) spectrum of compound **10**.

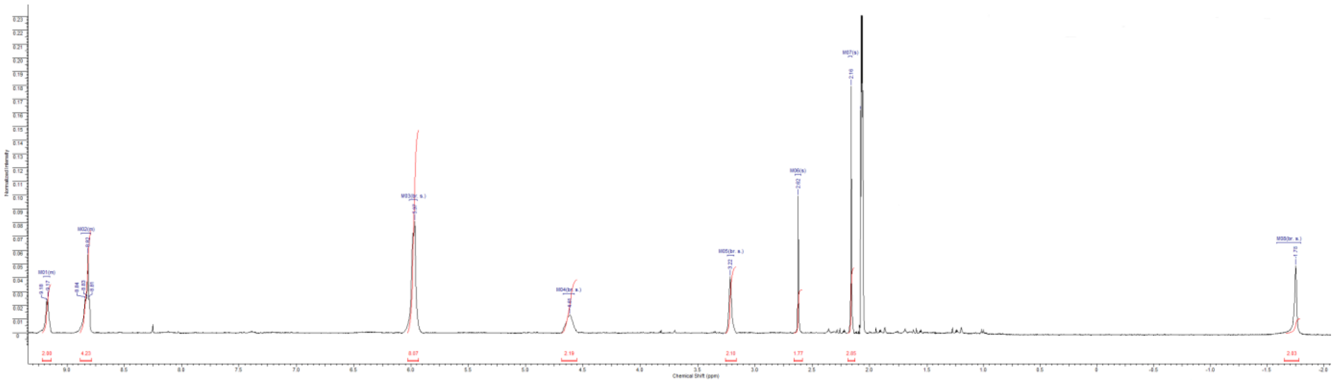

Figure S21 – ^{1}H NMR (400 MHz, $(CD_3)_2CO$) spectrum of compound **11**.

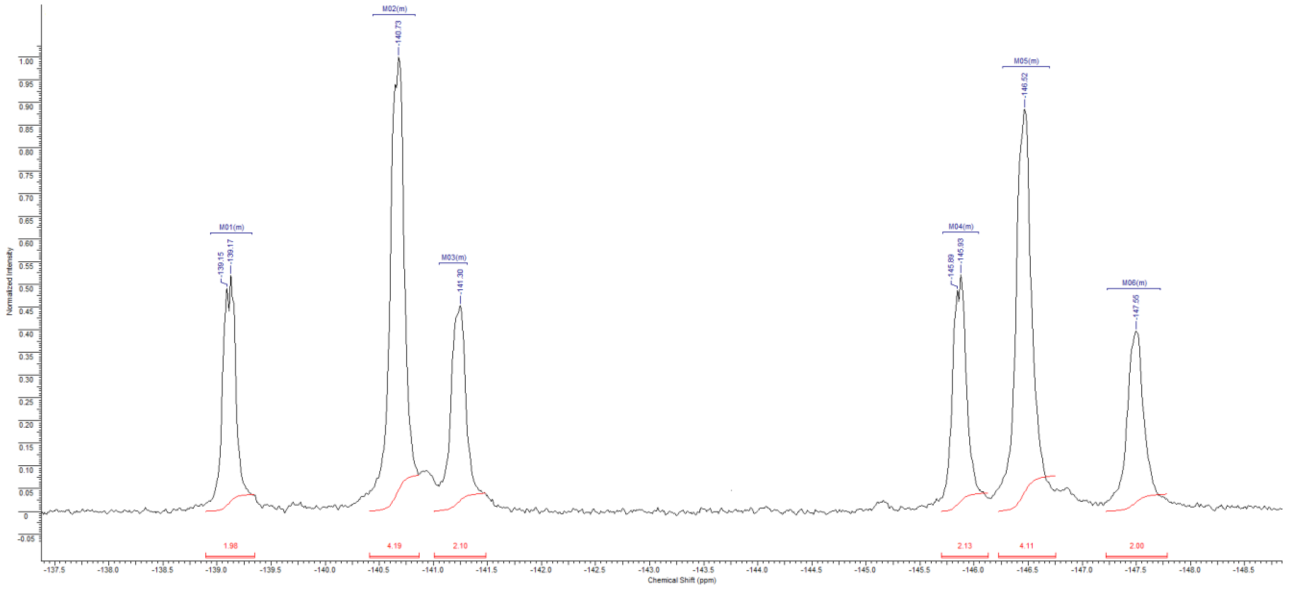

Figure S22 – ^{19}F NMR (376 MHz, $(CD_3)_2CO$) spectrum of compound **11**.

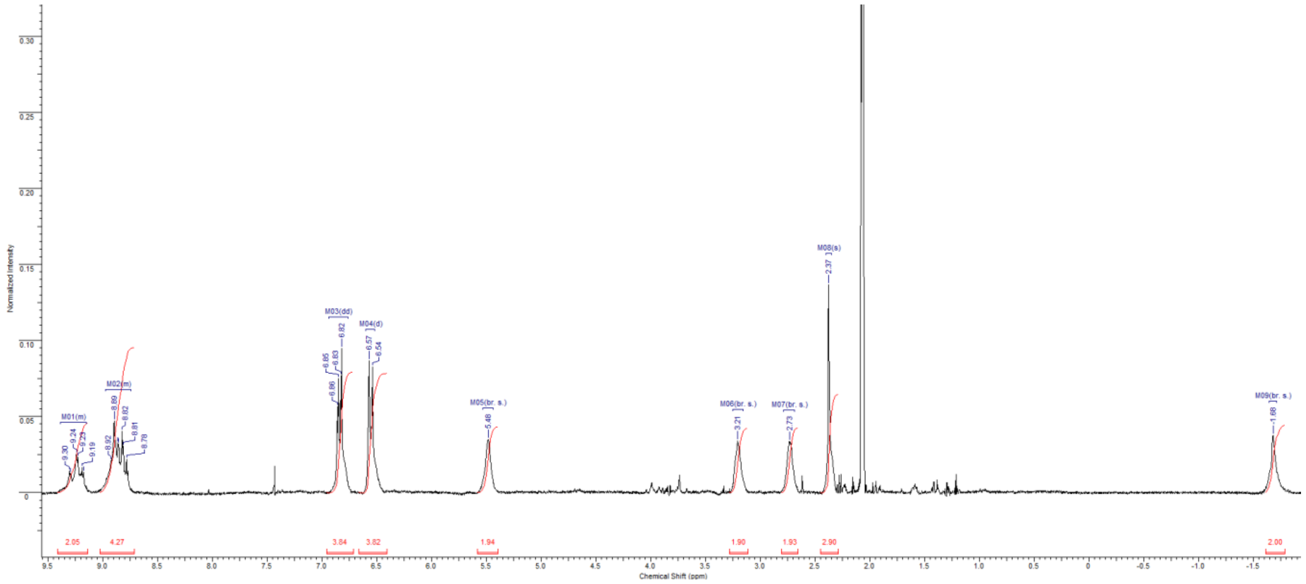

Figure S23 – ^{1}H NMR (400 MHz, $(CD_3)_2CO$) spectrum of compound **12**.

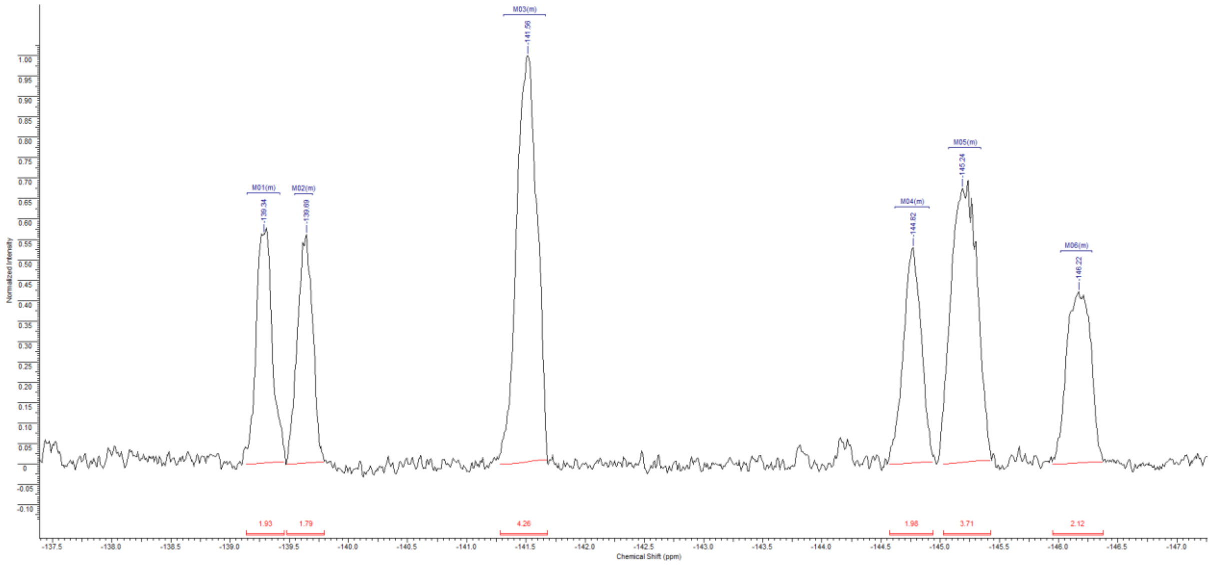

Figure S24 – ^{19}F NMR (376 MHz, $(CD_3)_2CO$) spectrum of compound **12**.

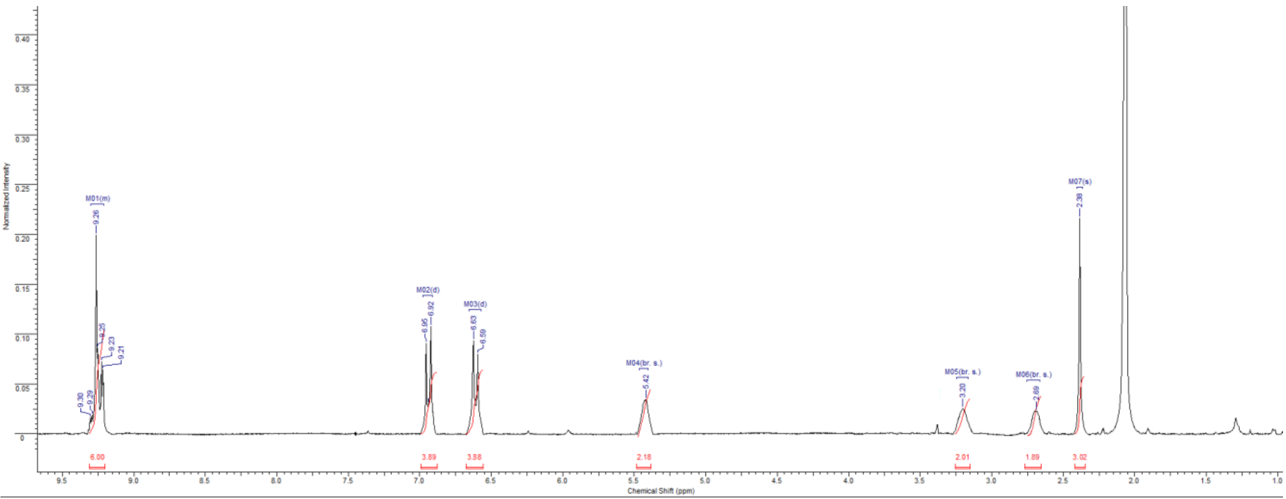

Figure S25 – ^{1}H NMR (400 MHz, $(CD_3)_2CO$) spectrum of compound **13**.

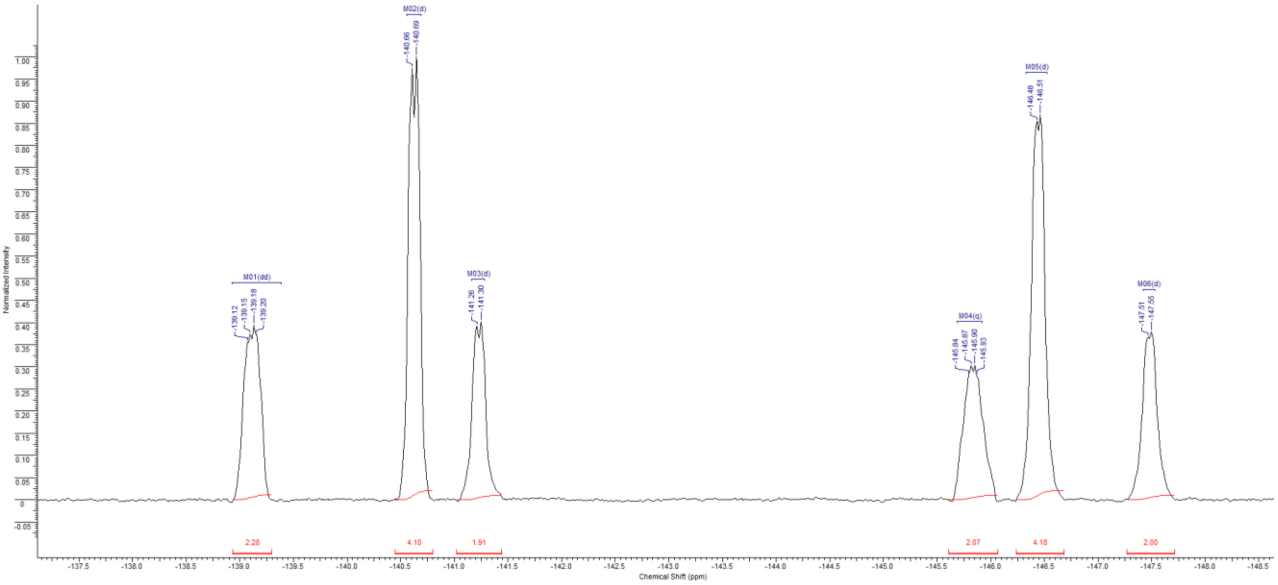

Figure S26 – ^{19}F NMR (376 MHz, $(CD_3)_2CO$) spectrum of compound **13**.

Mass-spectra

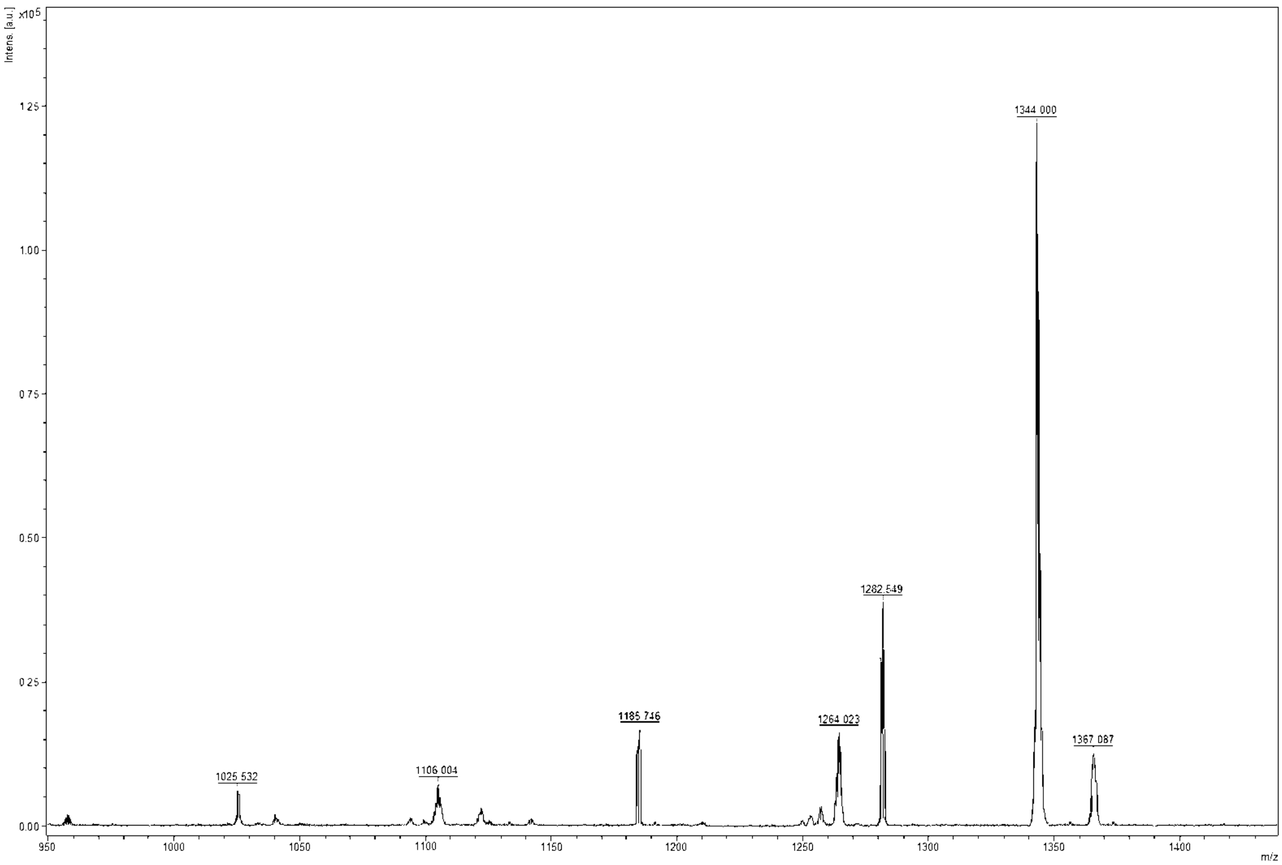

Figure S27 – mass-spectrum (MALDI) of compound **5a**

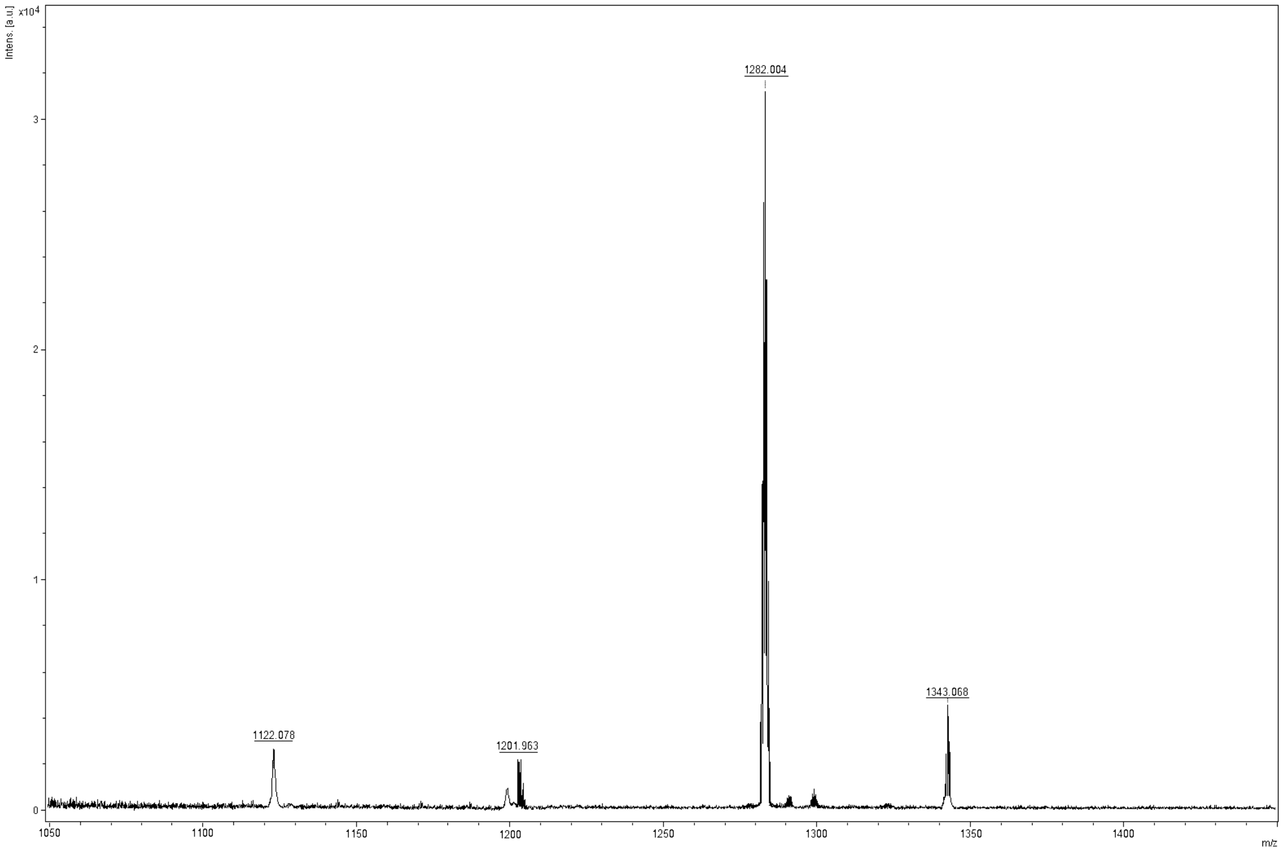

Figure S28 – mass-spectrum (MALDI) of compound **6**

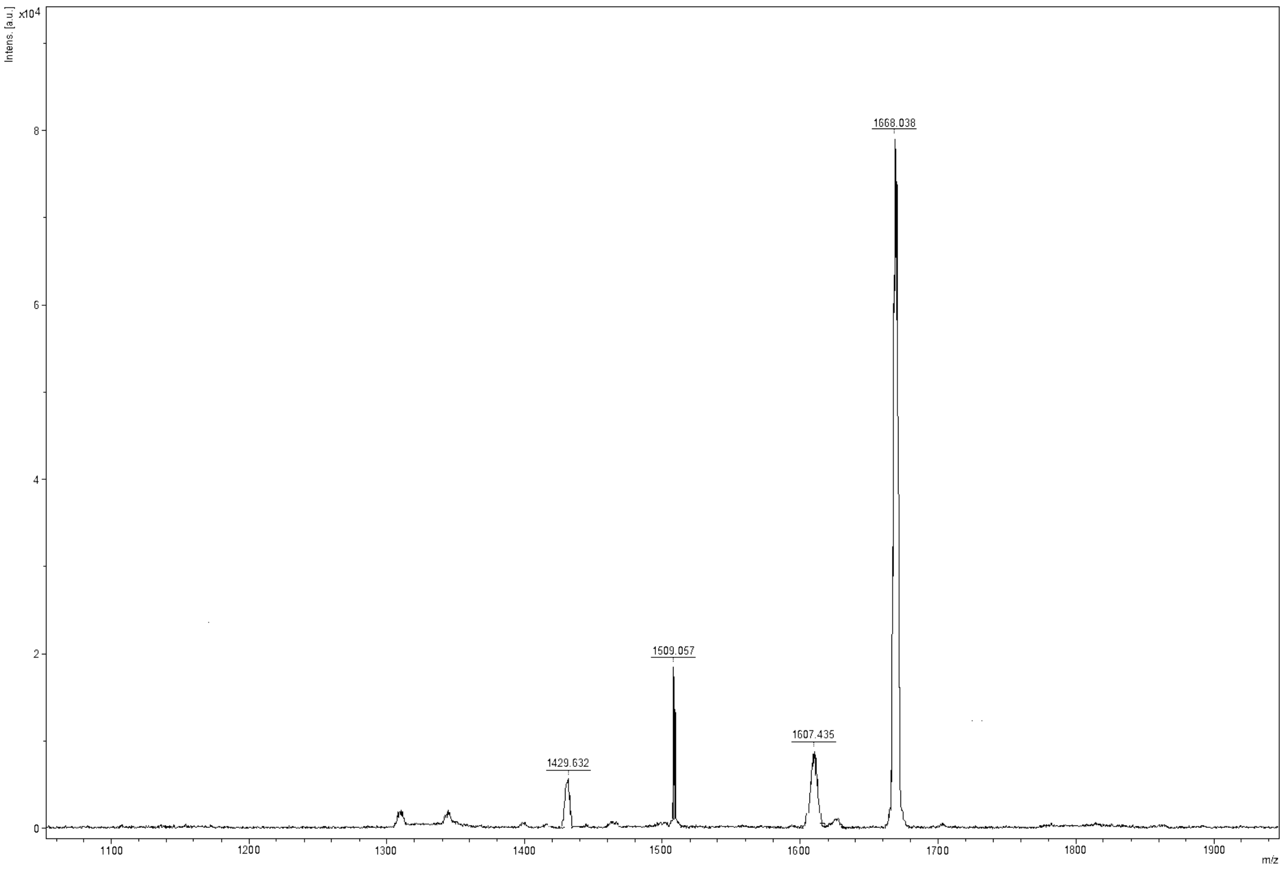

Figure S29 – mass-spectrum (MALDI) of compound **7a**

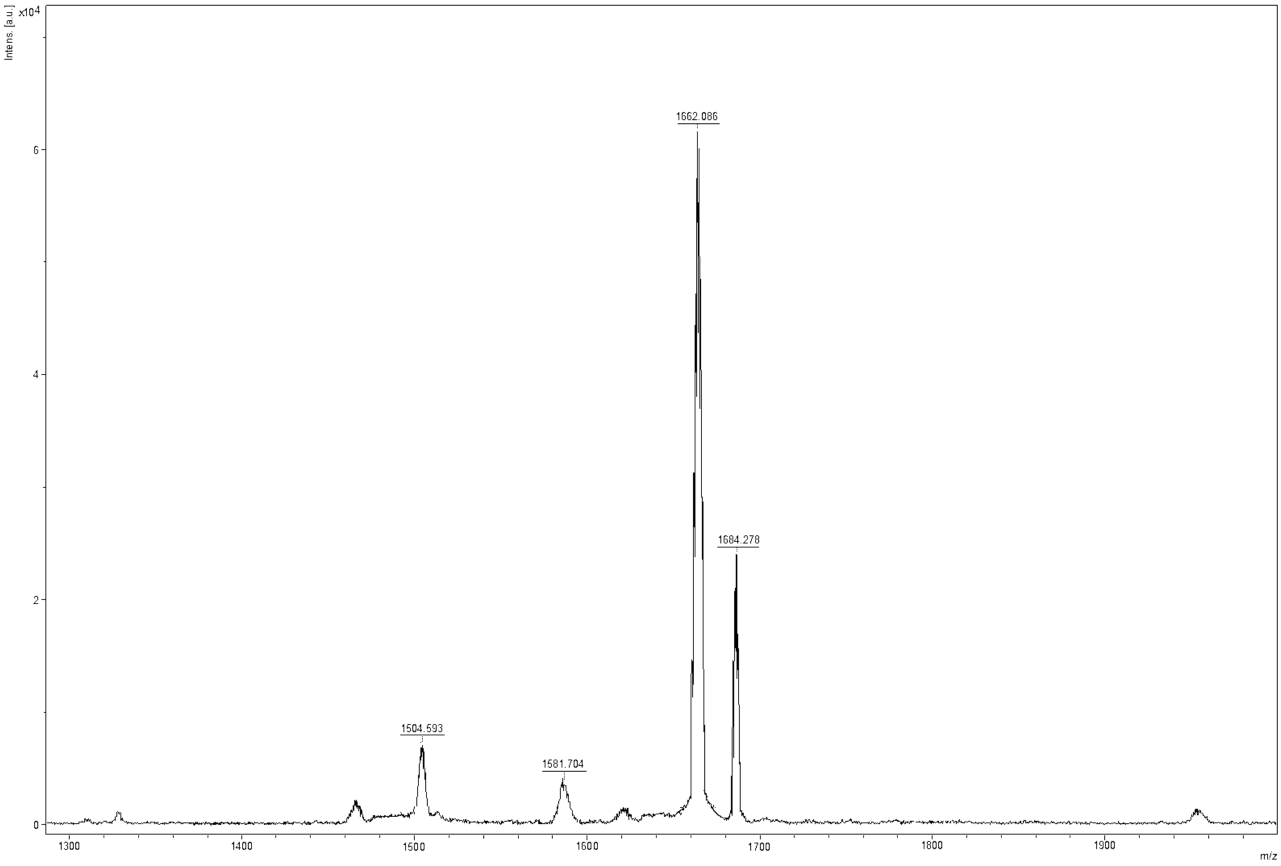

Figure S30 – mass-spectrum (MALDI) of compound **7b**

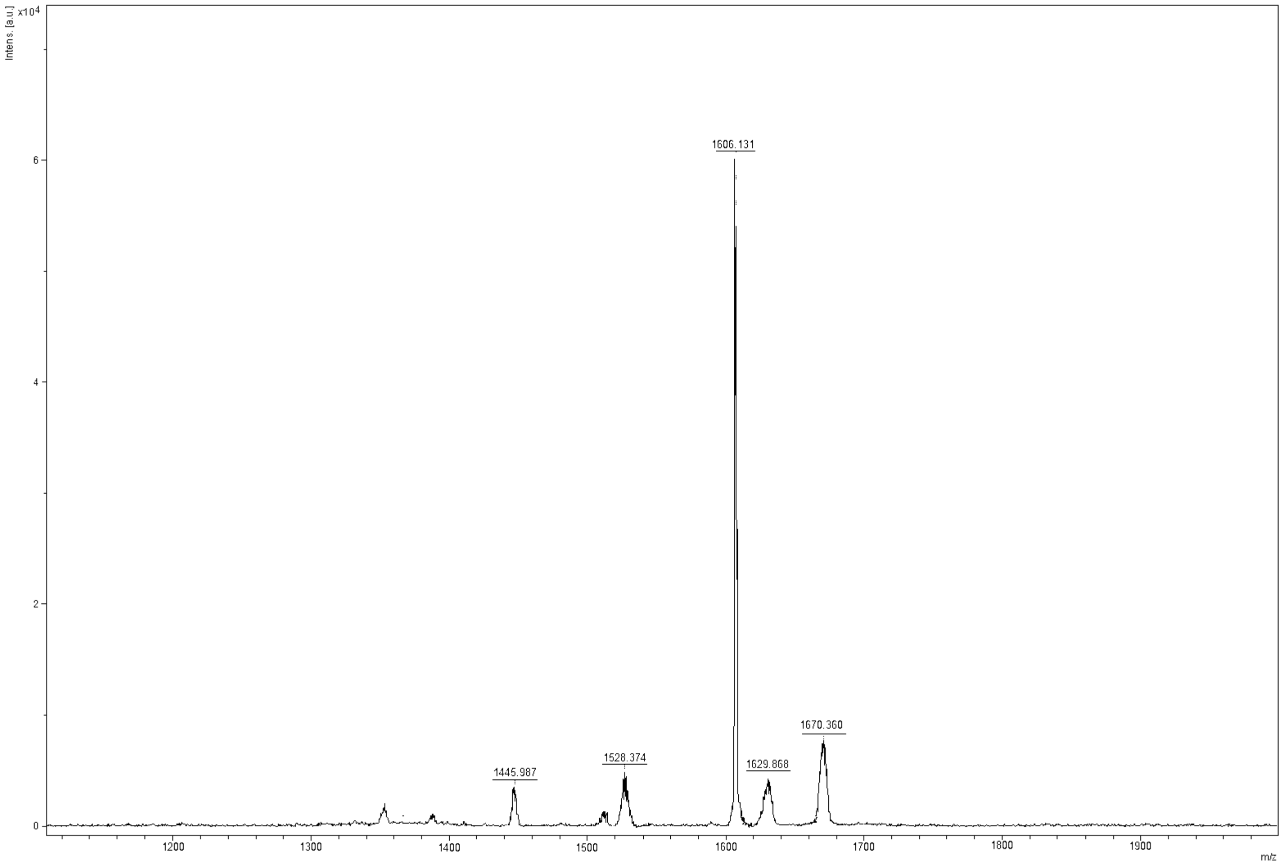

Figure S31 – mass-spectrum (MALDI) of compound **8**

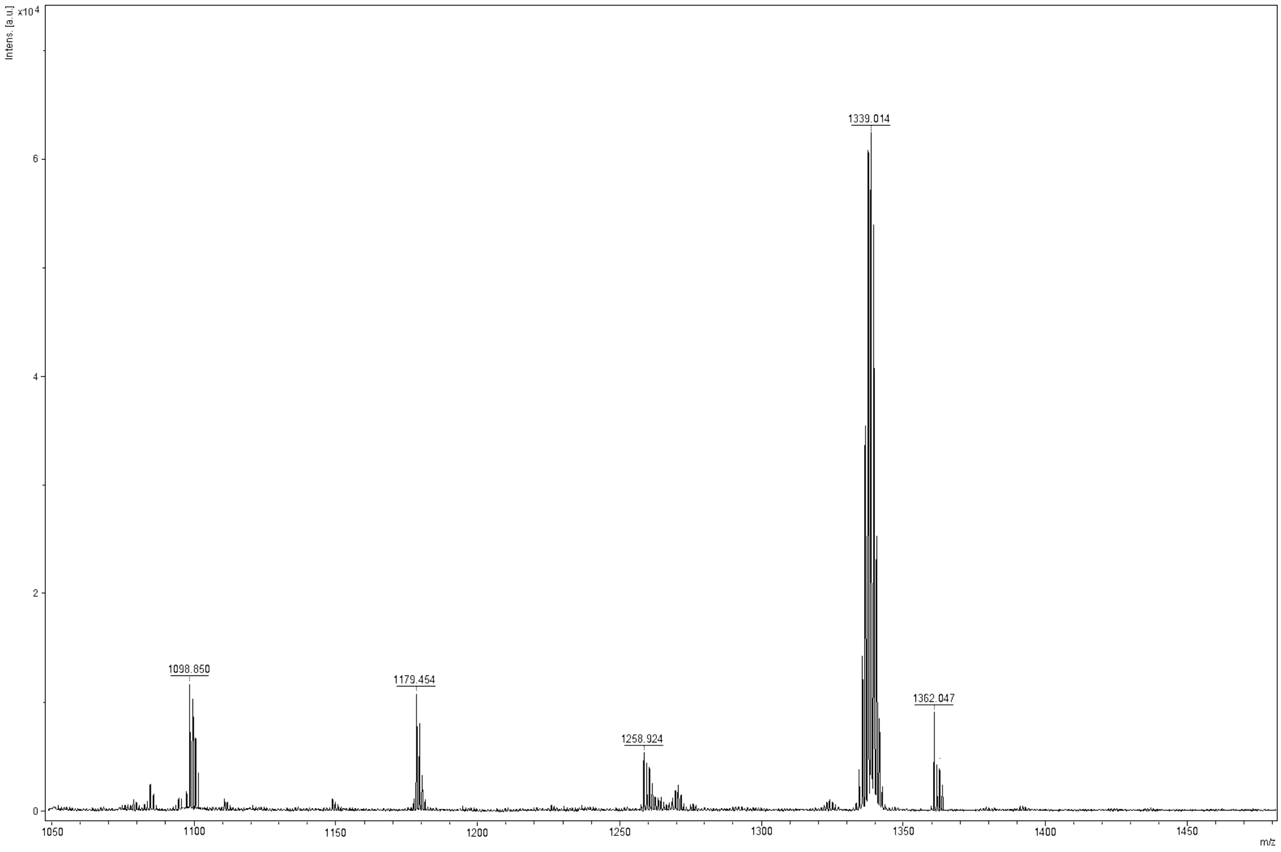

Figure S32 – mass-spectrum (MALDI) of compound **12**

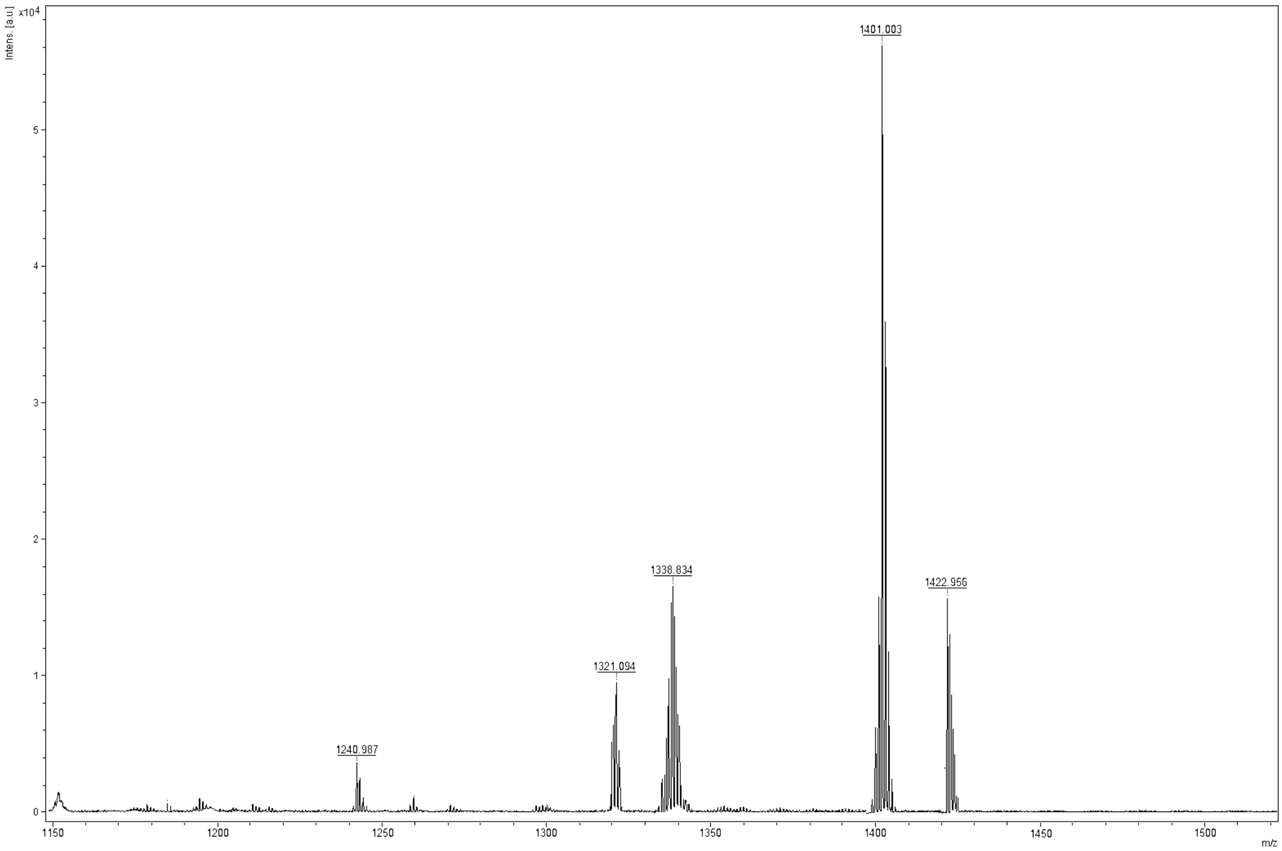

Figure S33 – mass-spectrum (MALDI) of compound **13**
